# Novel Acyl Phosphine Oxides as Low-Migration Type I Photoinitiators for Radical Polymerization

**DOI:** 10.3390/polym18212720

**Published:** 2026-11-02

**Authors:** Yinping Wu, Jiayun Zhong, Chunliu He, Jiaxin Huang, Lirong Zhang, Bo Zhang, Xiang Li

**Affiliations:** 1 School of Environmental and Chemical Engineering, Zhaoqing University, Zhaoqing 526061, China; 14718183222@163.com (J.Z.); a18933507736@163.com (C.H.); 13802893774@163.com (L.Z.); 2 New Energy and New Materials Research Center, Zhaoqing University, Zhaoqing 526061, China

**Keywords:** acylphosphine oxide, good solubility, liquid, low migration, photopolymerization

## Abstract

TPO-L (Ethyl(2,4,6-trimethylbenzoyl) phenylphosphinate) is a liquid high-efficiency photoinitiator, which is widely used in the UV-LED photopolymerization reaction field. After TPO-L participates in the photopolymerization reaction, the TPO-L that did not participate in the reaction will migrate to the surface of a coating, causing the coating surface to turn yellow and accelerating the aging process of the coating. This issue has attracted increasing attention. To address the high migration issue of TPO-L, in this experiment, three new type I photoinitiators were designed and synthesized, namely TPOLs with acylphosphine oxide structures. The structure of TPOLs can be modified by different lengths of methyl-terminal ethoxy chains. The properties of TPOLs were systematically evaluated through migration experiments, monomer solubility tests, absorption spectra, photopolymerization kinetics, photolysis, free radical trapping experiments, and cellular cytotoxicity. The results show that the absorption maxima of the TPOLs are located at around 372 nm. TPOLs have good solubility in acrylic monomers, and TPOL-2 and TPOL-3 in particular exhibit high photopolymerization efficiency. At a concentration of 50 μM, the cell survival rates of TPOL-1 and TPOL-3 are approximately 90%. It is noteworthy that the migration rate of TPOL-3 in the cured film is as low as 0.02%, significantly lower than the 0.98% of TPO-L, indicating that TPOL-3 has higher migration stability and better environmental compatibility. In summary, these results indicate that TPOL-3 has high initiation efficiency during LED curing, good solubility in monomers, and a low migration rate, making it a promising candidate material for low-migration photopolymerization applications.

## 1. Introduction

Photopolymerization technology enables liquid monomer formulations to rapidly be converted into a solid polymer after absorbing light energy [1]. Compared with traditional thermal polymerization, photopolymerization technology has several advantages, including faster curing speed, the lack of need for solvent processing (thus avoiding the emission of volatile organic compounds) and lower energy consumption [2,3]. Photopolymerization technology has made rapid progress in industrial applications, such as in the fields of adhesives [4], lithography [5,6,7], inks [8,9,10], and coatings [11,12,13], mainly due to its unique spatiotemporal scalability. Moreover, this technology assumes an indispensable role in high-end applications, for instance, 4D printing [14,15,16] and UV-LED photocurable coatings [17,18,19]. Photopolymerization systems typically consist of monomers, photoinitiators (PIs), coinitiators, and so on. Although PIs typically constitute only 1–8% of the photocurable formulation, they represent the most critical component, since their photosensitivity governs the polymerization rate and efficiency as well as the wavelength of the light source required for curing. Based on the active species produced, photopolymerization falls into two categories: the free radical type and cationic type. Applied research mainly focuses on the former, whereas conventional free radical PIs are classified into type I (photoinduced α-cleavage) and type II (hydrogen abstraction from a donor by the excited state) based on their distinct generation pathways [20,21]. Type I photoinitiators generate free radicals via bond cleavage; acylphosphine oxides are a typical example. In contrast, type II photoinitiators, such as thioxanthone derivatives, need a hydrogen donor to participate in the radical generation process.

As LED light sources advance rapidly, the emergence of novel photoinitiators has become essential to enable the fast curing of acrylate monomers upon LED exposure. Due to the fact that type I acylphosphine photoinitiators show high absorption in the ultraviolet wavelength range of 350 to 390 nm, and the free radicals generated after decomposition have high initiation efficiency, they have attracted extensive attention from the academic community. Currently, the commercially available photoinitiators based on acyl oxides include TPO, TPO-L and BAPO, all of which are acylphosphine oxides with a 2,4,6-trimethylbenzoyl structure [22]. TPO-L is widely used due to its good solubility and rapid photopolymerization rate. However, as a small molecule photoinitiator, the residual broken fragments and unreacted molecules of TPO-L in the cured coating may migrate to the coating surface, causing the coating surface to turn yellow and accelerating the aging process of the coating. As a result, the service life of the coating is shortened. Therefore, many researchers are dedicated to exploring photoinitiators that have strong absorption properties under LED light sources, enhance light polymerization efficiency, and reduce the migration rate. Acylphosphine oxides display favorable absorbance in the 350–390 nm region, which aligns well with the emission spectrum of UV-LEDs. Therefore, much effort has been directed toward developing new APOs for use as type I radical photoinitiators.

Recently, the migration of unreacted photoinitiators and their degradation products has attracted the attention of many scientists [23,24,25]. Regarding the problem of the high mobility of photoinitiators, two solutions have been developed to address this issue. One of the strategies is to increase molecular weight, for example, by preparing high-molecular-weight photoinitiators. Zhao et al. [26] developed two novel organosilicon-bearing APO photoinitiators, TPO-Si and TPO-L-Si, linked via thiol bridges, with molecular masses of 866 and 834, respectively, which contributes to their enhanced thermal stability and mobility. Qu et al. [27] developed two triphenylamine- and pyrene-functionalized acylphosphine oxide photoinitiators (NHPO and BHPO), whose absorption maxima are redshifted to 397 and 419 nm, respectively, and NHPO exhibits good biocompatibility and low mobility (only 0.23%). Qu et al. [28] prepared four novel APO photoinitiators functionalized with a pyrimidinedione moiety, with molecular masses in the range of 441–618 and a migration rate of 0.22%.

Another approach is to incorporate polymerizable groups, and most recent studies have focused on introducing such groups. Our group [29,30,31] reported the preparation of three classes of acylphosphine oxide photoinitiators: (allyl)carbazolyl derivatives, etheroxybenzoyl phosphine oxides (absorbing at 350–420 nm), and low-mobility type I APOs. The acylphosphine oxide chromophore was functionalized with amine [30] and vanillin [32]. These strategies were designed to improve the safety and performance of photopolymerization technology across a wide range of applications. Besides being interested in low-migration acylphosphine oxide photoinitiators, researchers have also synthesized and applied a number of novel acylphosphine oxide photoinitiators [22,33,34,35,36,37].

**Figure 1:**
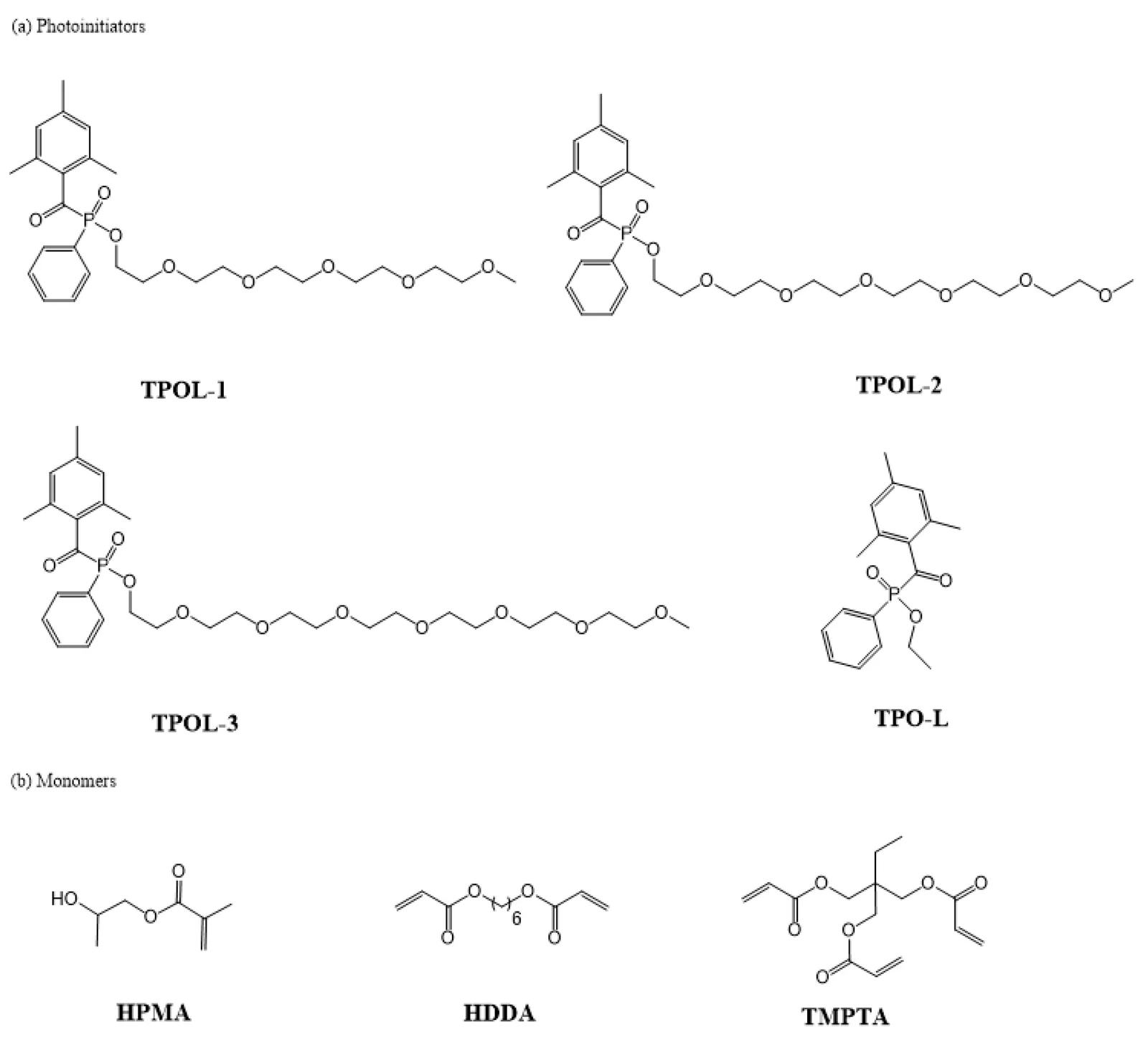
The molecular structures of photoinitiators and the monomers used in this study.

Structural modifications have been investigated by researchers to mitigate the migration tendency of small-molecule photoinitiators, in response to their strong odor and high mobility issues. In this work, three new acylphosphine oxide photoinitiators (TPOLs) were designed and synthesized, as depicted in Figure 1. We evaluated the performance of TPOLs and TPOL in acrylic monomer fiber-reinforced plastics and simultaneously conducted detailed studies on their migration stability, photophysical properties, photophysical properties, solubility, and cytotoxicity.

## 2. Materials and Methods

### 2.1. Materials

The preparation of phenyl(2,4,6-trimethylbenzoyl) phosphinic acid was carried out in our lab. All raw materials, solvents, and monomers used in the experiments were purchased from Energy Chemical (China). We designed and synthesized three acylphenylphosphine oxide photoinitiators (Figure 1).

### 2.2. Preparation of TPOLs (TPOL-1/TPOL-2/TPOL-3)

Step I: Oxalyl chloride (0.1635 g, 1.2882 mmol), 2 drops of DMF, dichloromethane (1.05 mL), and phenyl(2,4,6-trimethylbenzoyl)phosphonic acid (0.1530 g, 0.5307 mmol) were added to a 20 mL vial cooled in an ice bath. The mixture was stirred at 0 °C for 5 min in the dark and then at ambient temperature for 2 h. Volatile components (excess oxalyl chloride and DCM) were evaporated under vacuum, yielding a light brownish yellow oily residue that was carried forward without purification.

**Figure 2:**
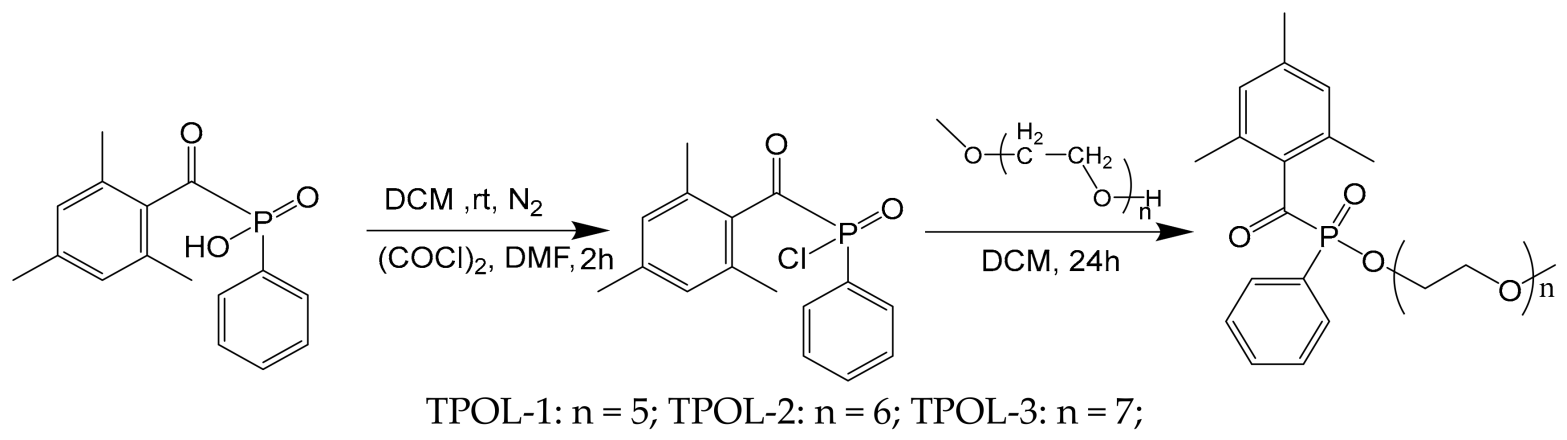
Synthetic route for TPOLs.

Step II: The crude product (0.5270 mmol) from the previous step was dissolved in 0.9 mL of DCM. An equimolar amount (0.5270 mmol) of hexaethylene glycol monomethyl ether (or heptaethylene/pentaethylene glycol monomethyl ether) was added. The solution was cooled to 0 °C. Triethylamine (0.5270 mmol) was then introduced. After 10 min, the ice bath was removed. The mixture was stirred uniformly in the dark at room temperature for 5 h. The reaction was stopped when thin-layer chromatography monitoring indicated that the raw materials had completely converted into the desired products. After quenching the reaction with distilled water, the pH value of the reaction mixture was adjusted to a slightly alkaline state using a weak base. The mixture was extracted with ethyl acetate more than three times until all the products were extracted. The mixed organic layer was dried to remove moisture. The solvent was removed under vacuum. The resulting pale yellow liquid was obtained after purification (Figure 2).

TPOL-1: HRMS (*m*/*z*): (APCI) calcd. for C_27_H_39_O_8_P [M]^−^: 522.2383, found 522.2379. ^31^P NMR (203 MHz, CDCl_3_) δ 18.12 (s). ^13^C NMR (126 MHz, CDCl_3_) δ 214.96 (*J* = 113.5 Hz, d), 139.87 (s), 136.40 (*J* = 47.4 Hz, d), 134.39 (s), 133.30 (*J* = 2.7 Hz, d), 132.89 (*J* = 9.9 Hz, d), 128.57 (*J* = 6.4 Hz, t), 127.29 (s), 126.30 (s), 71.82 (s), 70.71–70.27 (m), 70.09 (*J* = 5.8 Hz, d), 64.85 (*J* = 7.7 Hz, d), 58.91 (s), 21.10 (s), 19.32 (s). ^1^H NMR (500 MHz, CDCl_3_) δ 7.79 (*J* = 11.6, 7.3 Hz, 2H, dd), 7.55 (*J* = 10.9, 4.0 Hz, 1H, dd), 7.47–7.36 (2H, m), 6.75 (2H, s), 4.25–4.01 (2H, m), 3.63 (*J* = 5.0 Hz, 2H, t), 3.58 (*J* = 9.7 Hz, 10H, d), 3.52 (4H, s), 3.49–3.46 (2H, m), 3.31 (*J* = 4.6 Hz, 3H, d), 2.21 (3H, s), 2.09 (6H, s). Yellow viscous liquid. Yield: 44.99%.

TPOL-2: HRMS (*m*/*z*): (APCI) calcd. for C_29_H_43_O_9_P [M]^−^: 566.2645, found 566.2641. ^31^P NMR (203 MHz, CDCl_3_) δ 18.12 (s). ^13^C NMR (126 MHz, CDCl_3_) δ 215.02 (*J* = 113.4 Hz, d), 139.98 (s), 136.45 (*J* = 46.9 Hz, d), 134.49 (s), 133.38 (s), 132.99 (*J* = 10.0 Hz, d), 128.65 (*J* = 6.3 Hz, t), 127.35 (s), 126.36 (s), 71.92 (s), 71.06–69.35 (m), 64.93 (*J* = 7.6 Hz, d), 59.02 (s), 21.19 (s), 19.41 (s). ^1^H NMR (600 MHz, CDCl_3_) δ 8.01–7.75 (2H, m), 7.60 (*J* = 7.4 Hz, 1H, t), 7.52–7.43 (2H, m), 6.80 (2H, s), 4.28–4.09 (2H, m), 3.73–3.65 (2H, m), 3.66–3.60 (14H, m), 3.57 (*J* = 1.5 Hz, 4H, d), 3.55–3.51 (4H, m), 3.36 (3H, s), 2.26 (3H, s), 2.14 (6H, s). Yellow viscous liquid. Yield: 39.5%.

TPOL-3: HRMS (*m*/*z*): (APCI) calcd. for C_31_H_47_O_10_P [M]^−^: 610.2907, found 610.2901. ^31^P NMR (203 MHz, CDCl_3_) δ 18.08 (s). ^13^C NMR (126 MHz, CDCl_3_) δ 215.03 (*J* = 113.5 Hz, d), 139.97 (s), 136.47 (*J* = 47.0 Hz, d), 134.49 (s), 133.38 (*J* = 2.4 Hz, d), 132.99 (*J* = 9.9 Hz, d), 128.66 (*J* = 6.4 Hz, t), 127.37 (s), 126.39 (s), 71.93 (s), 70.54 (*J* = 7.7 Hz, d), 70.20 (*J* = 5.8 Hz, d), 64.94 (*J* = 7.7 Hz, d), 59.03 (s), 21.20 (s), 19.42 (s). ^1^H NMR (600 MHz, CDCl_3_) δ 7.82 (*J* = 11.5, 7.8 Hz, 2H, dd), 7.58 (*J* = 7.5 Hz, 1H, t), 7.46 (*J* = 7.46, 3.6 Hz, 2H, m), 6.78 (2H, s), 4.27–4.02 (2H, m), 3.66 (*J* = 6.7 Hz, 2H, t), 3.61 (*J* = 9.1, 6.8 Hz, 18H, dd), 3.55 (4H, s), 3.53–3.48 (2H, m), 3.34 (3H, s), 2.24 (3H, s), 2.12 (6H, s). Yellow viscous liquid. Yield: 55.25%.

## 3. Results and Discussion

### 3.1. Optical Properties of TPOLs

**Figure 3:**
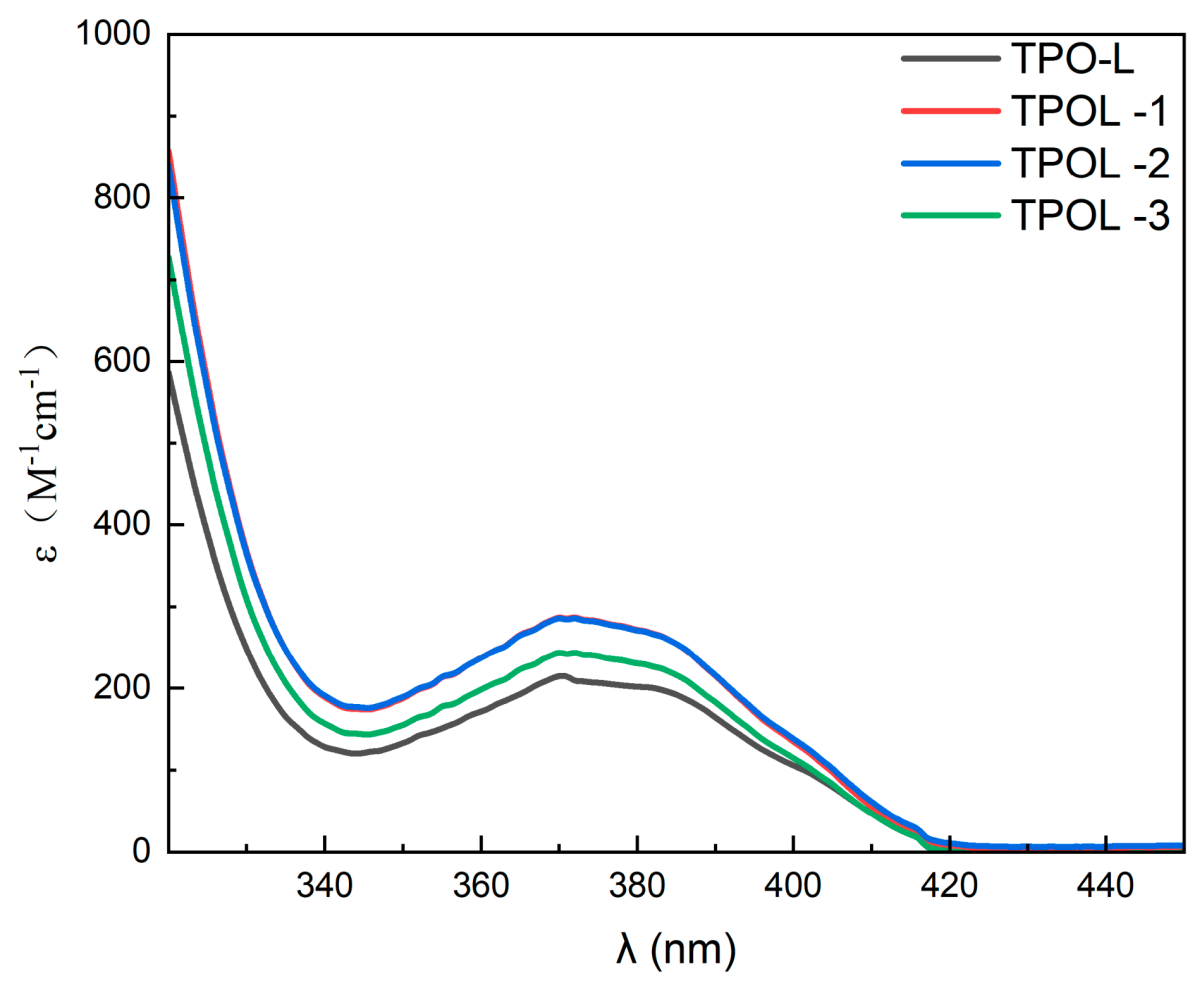
Absorption spectra of TPOLs and TPO-L in acetonitrile.

The UV absorption behaviors of the four photoinitiators (TPOL-1, TPOL-2, TPOL-3, and TPO-L) in CH_3_CN are illustrated in Figure 3 and Table 1 for comparative purposes. The maximum absorption values in the UV–visible absorption spectra of TPOL-1, TPOL-2 and TPOL-3 are all around 372 nm. Upon comparison, it was found that the molar absorption coefficients of TPOLs in the ultraviolet–visible region were all higher than those of TPO-L. The ε values of TPOL-1 and TPOL-2 at 372 nm reached 288 M^−1^cm^−1^, while that of TPOL-3 was 245 M^−1^cm^−1^. These findings suggest that increasing the molecular weight of acylphosphine oxide substituents improves absorptivity while leaving the absorption wavelength region unchanged.

**Figure 4:**
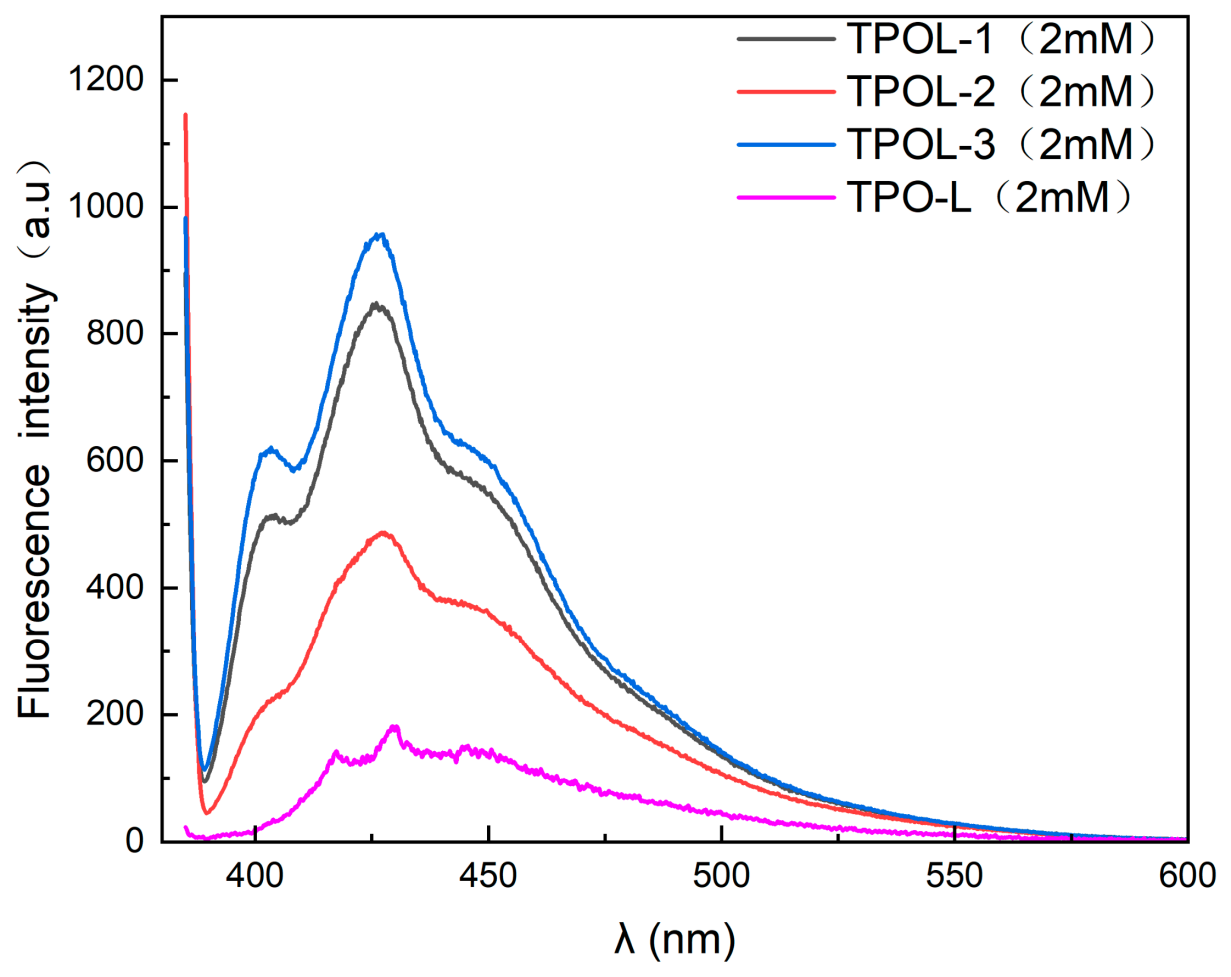
The fluorescence emission spectrum of TPOLs in acetonitrile (2 mM) with the excitation wavelength set at 380 nm.

The excited-state properties of TPOLs were investigated via an analysis of their fluorescence spectra (Figure 4 and Table 1). Relatively weak fluorescence intensities were observed for these compounds, indicating that a larger proportion of absorbed energy is consumed to produce free radicals. This feature facilitates the photopolymerization reaction.

Table 2 summarizes the calculated HOMO/LUMO energies of TPOLs. DFT analysis indicates that the lowest electronic transition is n→π* in nature, with both frontier orbitals mainly distributed over the phenyl (benzoyl)phosphine fragment—confirming that the Norrish type I photocleavage occurs at this moiety. TPOLs 1–3 differ in the length of their methyl-terminated ethoxy chains, yet their n→π* energy gaps (4.64, 4.70, and 4.70 eV) remain nearly constant, suggesting that the ethoxy chain length exerts minimal influence on the transition. The energy level difference Egap in TPO-L is 4.74 eV, which is close to that in TPOLs. The absorption range in the ultraviolet–visible absorption spectrum of TPOLs is also close to that of TPO-L, and there is no significant redshift in TPOLs.

### 3.2. Migration

The migration rate of TPOLs in the cured TMPTA film was evaluated through acetonitrile extraction experiments, and it was compared with TPO-L (Table 3). The significantly reduced migration of TPOLs relative to TPO-L can be further rationalized from kinetic perspectives. From a kinetic standpoint, the higher molecular weights of TPOLs (521.38–610.29 g/mol versus 316.34 g/mol for TPO-L) considerably restrict their translational mobility within the polymer network after curing. In crosslinked acrylate systems, TPOLs possess a large molecular volume, which not only slows down their diffusion rate within the film but also promotes physical entanglement with growing acrylate polymer chains, thereby effectively anchoring the photoinitiators and their fragments in the photocured film. Furthermore, the methyl-terminated ethoxy groups were introduced into the molecular structure of TPOLs. The methyl-terminated ethoxy groups promoted the reduction in molecular migration through two synergistic effects. Firstly, they increased the overall polarity of the molecules, enhancing the intermolecular interactions with polar monomers or oligomers; secondly, the flexible methyl-terminated ethoxy spacer segments facilitated conformational adaptation, thereby improving the compatibility of the photoinitiator with the membrane and reducing the tendency of phase separation, which is often a precursor to the migration phenomenon. From the migration rate results, it can be seen that TPOL-3 has the longest ethoxy chain segment among the three derivatives, and its extraction rate is the lowest (0.02%), which confirms the positive role of the ethoxy functional group in inhibiting migration. The above results are in agreement with the previously observed structure–migration relationships of other acylphosphine oxide derivatives [31]. Compared with TPO-L (0.98%), the migration rate values of TPOL-1, TPOL-2, and TPOL-3 (0.70%, 0.14%, and 0.02% respectively) all provide a considerable safety margin. The experimental results demonstrate that by introducing polar ethoxy groups into the acylphosphine oxide, increasing the molecular weight of the photoinitiator can significantly reduce the mobility data of the photoinitiator without compromising its photochemical properties. Therefore, TPOLs can be used as a potential alternative to TPO-L in industrial applications such as inks, adhesives, and coatings where low migration requirements are necessary.

### 3.3. Solubility

In this experiment, the solubility properties of TPOLs and TPO-L in three representative monomers—trimethylolpropane triacrylate (TMPTA), 1,6-hexanediol diacrylate (HDDA), and 2-hydroxypropyl methacrylate (HPMA)—were tested (Table 4). The solubility of TPOLs in the liquid monomer is comparable to that of TPO-L, and both can achieve mutual dissolution with the monomer. They all exhibit good compatibility. The similarity in solubility can be explained by the structural analogy between TPOLs and TPO-L. The different-length methyl-terminated ethoxy-substituted molecular structures introduced by the phosphorus groups in TPOLs do not significantly alter the overall polarity and crystallinity of the molecules, thus maintaining their solubility in methacrylate monomers. From an industrial application perspective, the good solubility of photoinitiators ensures their uniform dispersion within the resin matrix, which is a prerequisite for achieving consistent photopolymerization kinetics and the final material properties. Due to the wide application of TPO-L in commercial UV-curable coatings, inks and adhesives, the similar solubility properties of TPOLs make them an ideal low-migration alternative for TPO-L, facilitating the transition of TPOLs from laboratory-scale synthesis to industrial applications without the need to modify the addition method of photoinitiators in existing industrial application formulations. In conclusion, from the perspective of solubility, the newly synthesized TPOLs are a completely feasible alternative to TPO-L and provide the same level of ease of use when preparing photopolymerization application formulations.

### 3.4. Photochemical and ESR-ST

**Figure 5:**
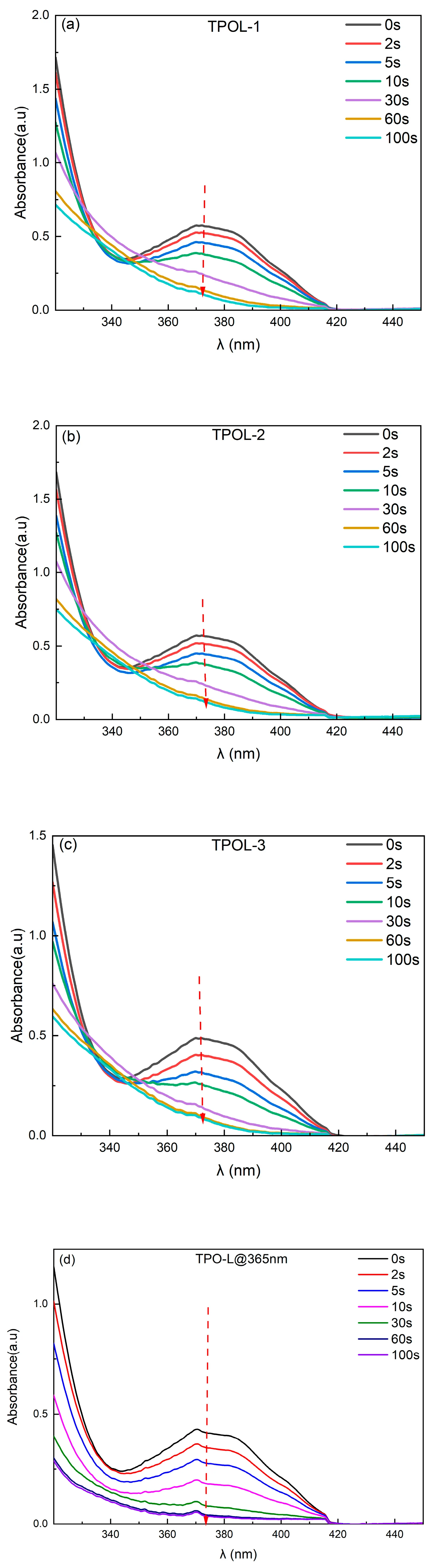
The steady-state photolysis reactions of TPOL1-3 and TPO-L were recorded under LED@365 nm with a photoinitiator concentration of 2 mM in acetonitrile, and the ultraviolet–visible spectra were obtained. (**a**) TPOL-1; (**b**) TPOL-2; (**c**) TPOL-3; (**d**) TPO-L.

Figure 5 shows the change in the absorption spectrum of the photoinitiator dissolved in CH_3_CN under irradiation with a 365 nm LED. TPOLs and TPO-L exhibited a significant decrease in absorbance within the wavelength range of 340–410 nm as the exposure time of the 365 nm light source increased, indicating that these substances undergo photodegradation reactions under light irradiation. The attenuation of the absorbance of the photoinitiator is consistent with the Norrish type I photocatalytic cleavage of the acyl–phosphorus bond, and it is also a typical reaction of the photodegradation of acylphosphine oxide photoinitiators. After absorbing light energy, acylphosphine undergoes α-cleavage, generating phosphoyl radicals and benzoyl-type radicals, both of which can serve as starting materials for vinyl polymerization reactions (Figure S1). During the photodegradation process, the synchronous decrease in the characteristic absorption bands of TPOLs indicates that the parent chromophore has been consumed, while the slight shift in the absorption spectrum may be attributed to the formation of new photoproducts. The above results confirm that all three types of TPOLs can absorb light energy and undergo efficient photodegradation under LED@365 nm, which is a necessary condition for them to act as free radical photoinitiators.

**Figure 6:**
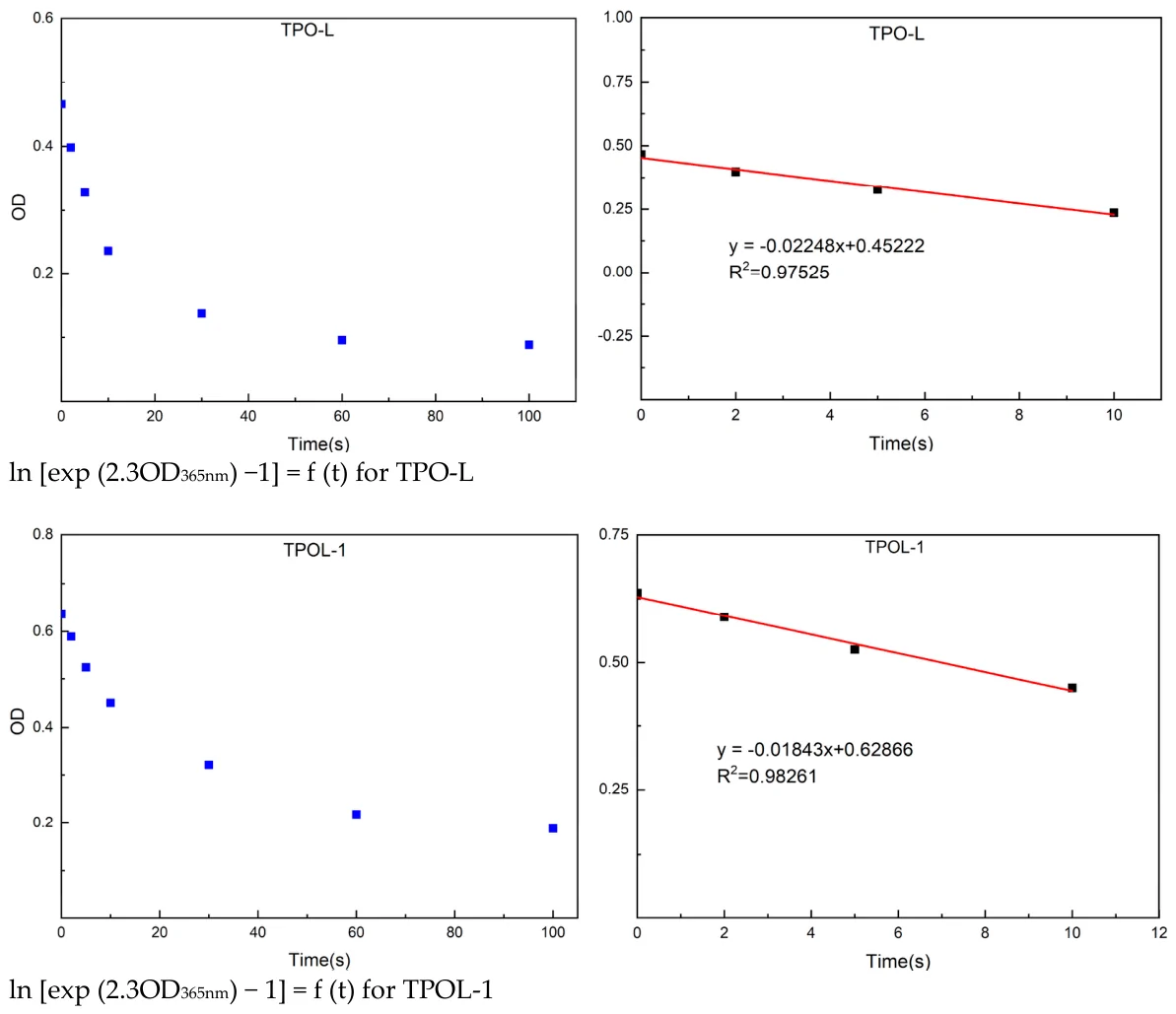

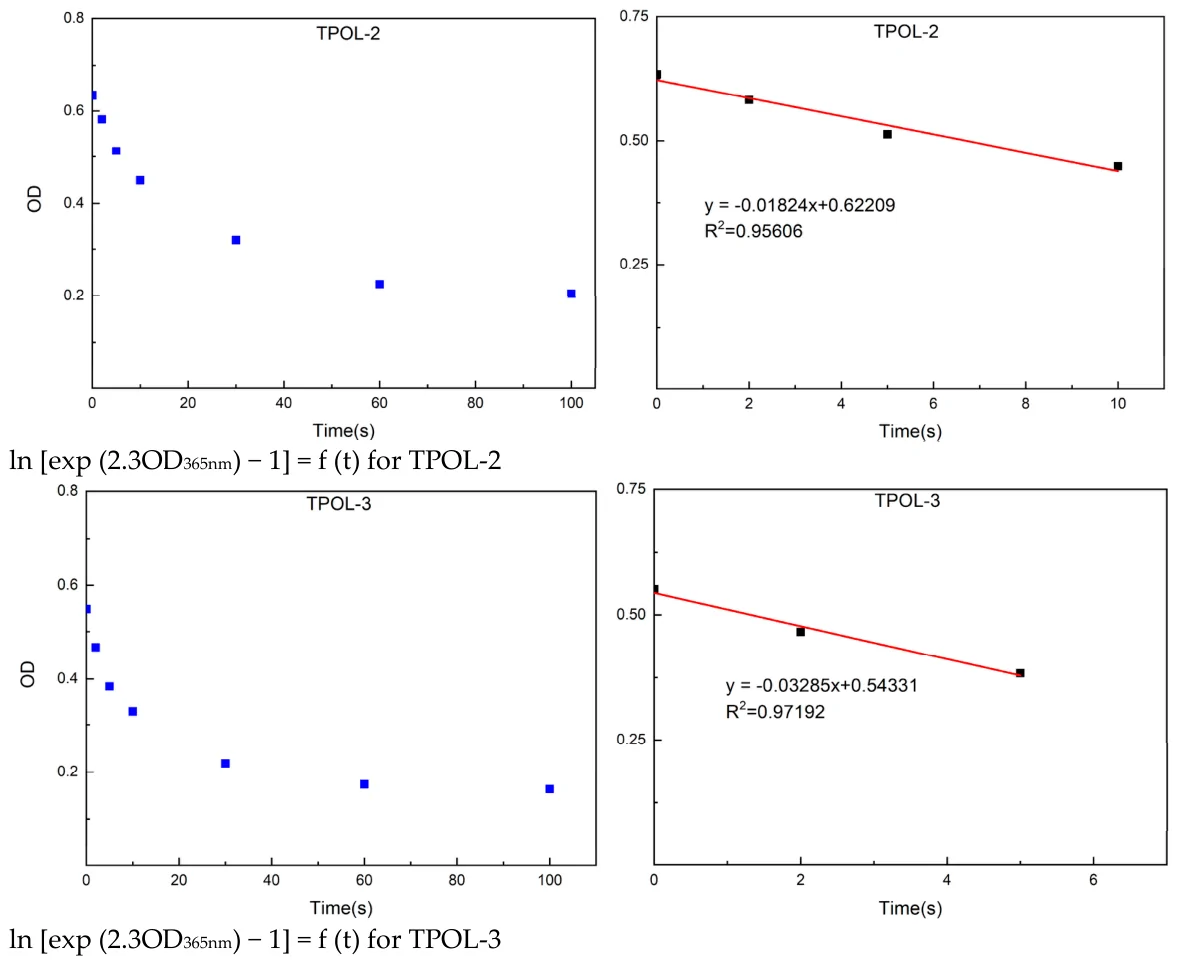
The slope of the function ln [exp(2.3OD_365nm_) − 1] = f(t) at the start of the reaction (−0.02248 for TPO-L, −0.01843 for TPOL-1, −0.01824 for TPOL-2, −0.03285 for TPOL-3).

The cleavage reaction was investigated by determining the quantum yield (ϕ), defined as the number of molecules undergoing α-cleavage per absorbed photon, for photolysis in acetonitrile. As shown in Figure 5, Figure 6 and Table 1, TPOL-3 exhibited the highest quantum yield (ϕ = 0.15), comparable to that of TPO-L (ϕ = 0.12), whereas TPOL-1 and TPOL-2 both gave a value of 0.07. Despite their lower quantum yields, TPOL-1 and TPOL-2 showed stronger absorption capacity than TPO-L, making them worthy of further investigation. Ultimately, only polymerization experiments can reveal the actual efficiency of these acylphosphine oxides and distinguish their photoinitiation behavior.

**Figure 7:**
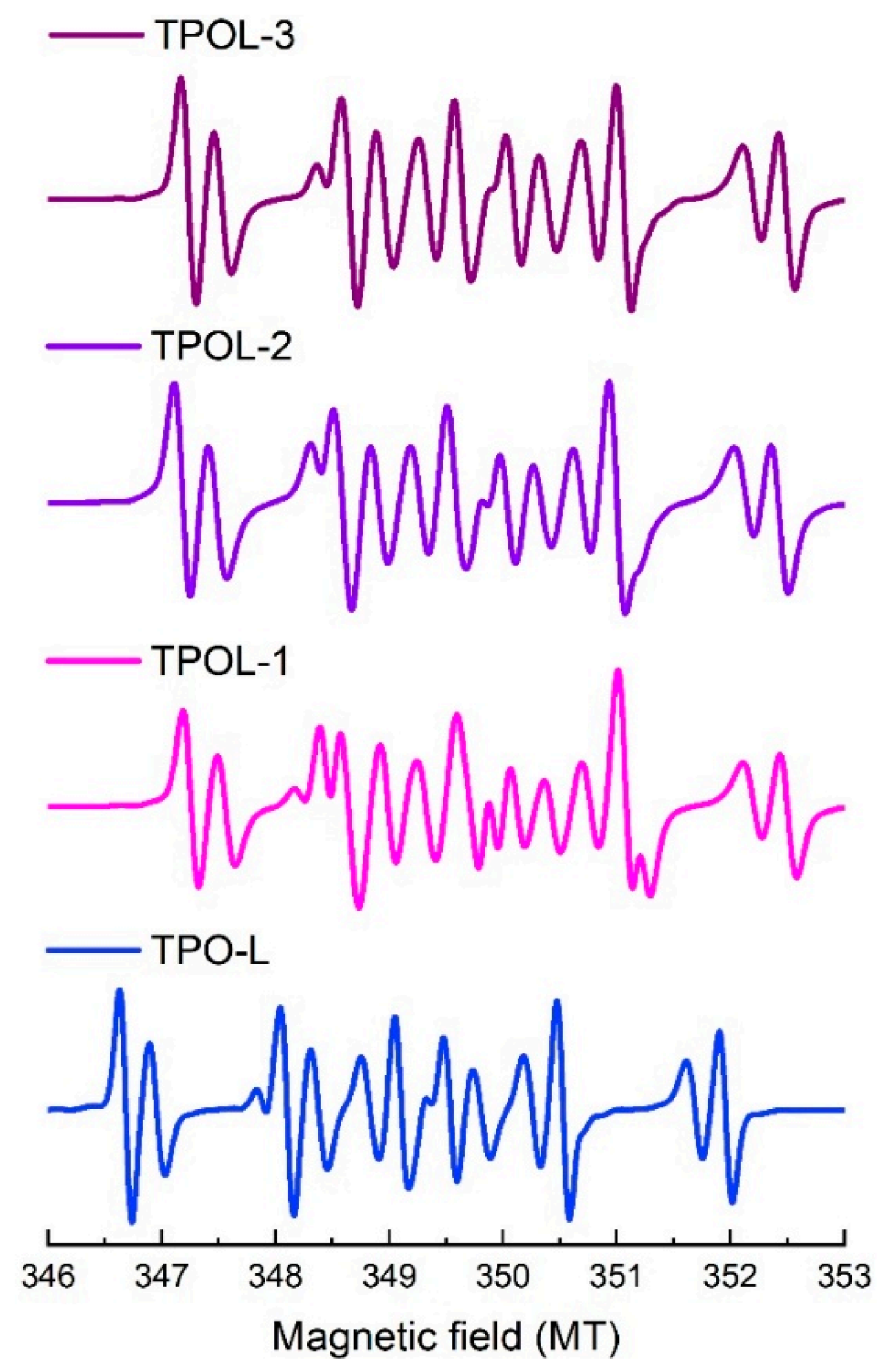
ESR-ST spectra of TPOL1-3 and TPO-L obtained after exposing photoinitiator/tert-butylbenzene to LED@365 nm irradiation for 60 s. Sample solutions composed of 1 mM photoinitiator and 0.01 M PBN dissolved in tert-butylbenzene.

We compared the hyperfine coupling constants (hfccs) of the phosphorus–oxygen free radicals produced by the cleavage of TPO-L and TPOLs. The results showed that the curves and values of their radical complexes were respectively close, confirming that the captured radical adducts have similarities in structure or conformation (Figure 7). Representative values were obtained for TPO-L (α_P_ = 2.16 MT; α_H_ = 0.28 MT; α_N_ = 1.43 MT) and TPOL-3 (α_P_ = 2.11; α_H_ = 0.29; α_N_ = 1.42 MT), which are in good agreement with previously reported hfccs for acylphosphine oxide-derived phosphono radicals. In conclusion, the ESR results clearly demonstrated that after the TPOLs and TPO-L were cleaved under the irradiation of a 365 nm light source, phosphorus–oxygen free radicals were generated, confirming that the TPOLs were subjected to the Norrish type I photolysis mechanism. Combined with the photodegradation phenomenon observed in the UV-vis spectra, these spin-trapping data firmly establish that all synthesized TPOLs and TPO-L undergo efficient radical generation, a prerequisite for their use as photoinitiators in polymerization reactions.

### 3.5. Photopolymerizations

**Figure 8:**
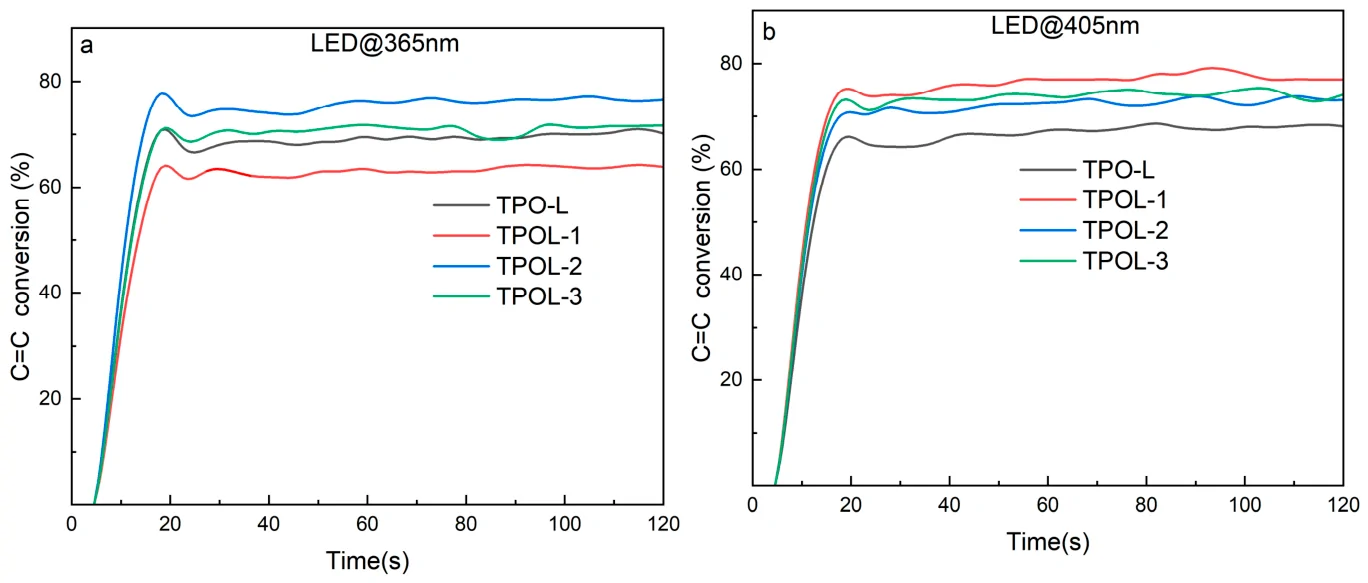
Conversion profiles of PIs in TMPTA (3.16 mmol PI/100 g TMPTA): (**a**) TMPTA under LED@365 nm; (**b**) TMPTA under LED@405 nm.

**Figure 9:**
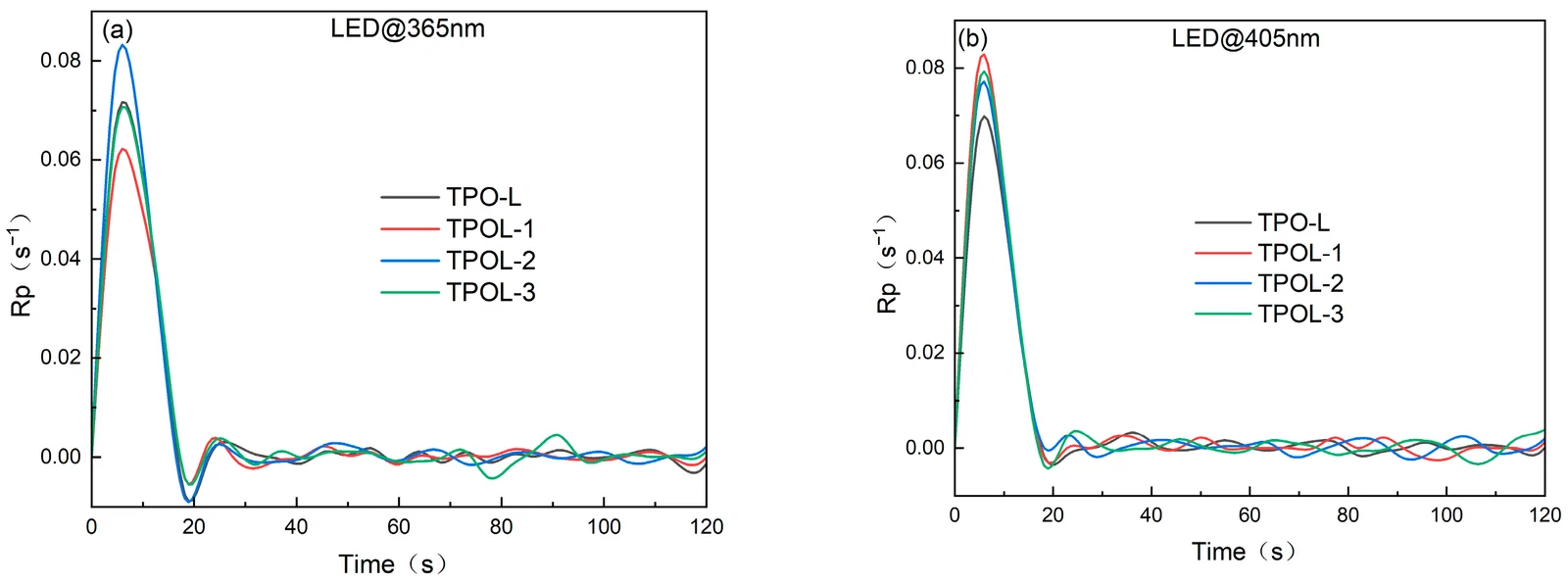
The photopolymerization rate of PIs (3.16 mmol PI/100 g TMPTA): (**a**) TMPTA under LED@365 nm; (**b**) TMPTA under LED@405 nm.

In order to study the photoinitiation capabilities of different photoinitiators at the same molar concentration (3.16 mmol PI/100 g TMPTA), the TPOL photoinitiators were investigated, and their photoinitiation capabilities under LED light sources of 365 nm and 405 nm were compared. As shown in Figure 8 and Figure 9, all the compounds exhibited polymerization reactions upon irradiation. In 100 g of TMPTA, with 3.16 mmol of photoinitiator being compared, the results show that under the LED@365 nm condition, the order of the photoinitiation performance of DC_120_ is TPOL-2 system > TPOL-3 system > TPO-L system > TPOL-1 system, while under the LED@405 nm condition, the order of the photoinitiation performance of DC_120_ in TMPTA is TPOL-1 system > TPOL-3 system > TPOL-2 system > TPO-L system. Under the illumination of the 405 nm LED light source, the photoinitiation efficiency of TPOLs was higher than that of TPO-L. This result might be due to the fact that the molar extinction coefficient of TPOLs at 405 nm is higher than that of TPO-L. Although the molecular weight of TPOL-1 is relatively low, the DC rate of TPOL-1 is only higher than that of TPO-L under LED@405 nm illumination. Therefore, molecular weight does not have a direct impact on the double bond conversion rate. As shown in Figure 8, under LED@365 nm and 405 nm irradiation, TPOL-2 and TPOL-3 exhibit higher photoinitiation efficiencies than TPO-L, despite their higher molecular weights. As can be seen from Figure 9, under LED@365 nm and 405 nm, the photopolymerization reactions both ended around 20 s. Under LED@365 nm irradiation, the order of the polymerization rate of the photoinitiators is TPOL-2 system > TPO-L system ≈ TPOL-3 system > TPOL-1 system. Under LED@405 nm irradiation, the order of the polymerization rate of the photoinitiators was TPOL-1 system > TPOL-3 system ≈ TPOL-2 system > TPO-L system. Therefore, under the illumination of LED@365 nm and 405 nm, the photoinitiation rates of the photoinitiators TPOL-2 and TPOL-3 are both higher than that of TPO-L. By comparing the photoinitiation efficiencies of the photoinitiators at the same molar concentration, TPOL-3 and TPOL-2 can be used as substitutes for TPO-L in LED@365 nm, while TPOL-1, TPOL-2, and TPOL-3 can all be used as substitutes for TPO-L in LED@405 nm.

### 3.6. Cellular Cytotoxicity

**Figure 10:**
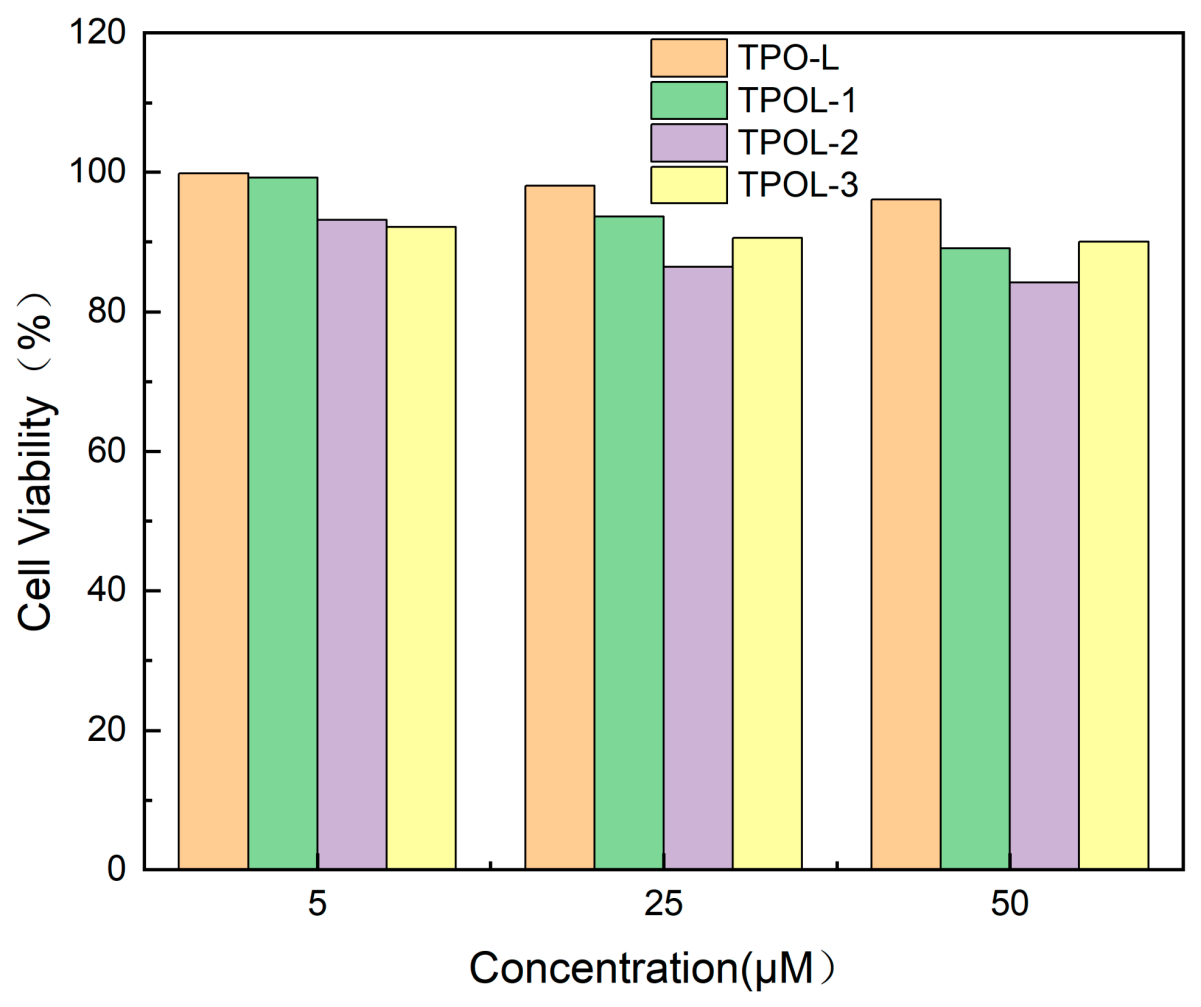
The cellular cytotoxicity of TPOLs and TPO-L after 24 h.

Cell compatibility is a crucial factor for the application of photoinitiators in the industrial field. To evaluate the biocompatibility of TPOL-1, TPOL-2, and TPOL-3, cytotoxicity assays were conducted comparing these compounds with TPO-L, as shown in Figure 10. Figure 10 indicates that at a concentration of 50 μM, the survival rate of L929 cells was higher than 80%, and the comparison results of cell compatibility were TPO-L > TPOL-1 > TPOL-3 > TPOL-2. At a 50 μM concentration of the photoinitiator, the cell survival rates of TPOL-1 and TPOL-3 were both higher than or close to 90%, while the cell survival rate of TPO-L was 96%, proving that TPOL-1 and TPOL-3 have superior biocompatibility. The above results indicate that TPOL-1 and TPOL-3 have broad prospects in industrial applications.

## 4. Conclusions

Three acylphosphine oxide photoinitiators were designed and successfully synthesized in the present work, namely TPOL1-3, which exhibit low migration and outstanding solubility in acrylate monomers. Compared with commercial TPO-L, the molar extinction coefficients of TPOL1-3 are higher. For TPOL-2 and TPOL-3, under the illumination of 365 nm and 405 nm light sources, photoinitiation efficiency is close to or higher than that of TPO-L, and the migration rate of TPOL-3 in the cured polymer is the lowest at 0.02%. In terms of cytotoxicity, at a concentration of 50 μM, the cell survival rates of TPOL-1 and TPOL-3 were approximately 90%. In terms of solubility in the monomer, TPOL1-3 and TPO-L are all miscible with the monomer. Therefore, the TPOL-3 developed in this study has low mobility, high photoinitiation efficiency, low cytotoxicity, and good solubility in monomers. TPOL-3 can be used as a substitute for TPO-L in low-mobility application fields.

## Figures and Tables

**Table 1:** Photophysical parameters of TPOLs.

PIs	λ_max_ (nm)	ε_max_ (Lmol^−1^cm^−1^)	ε_365_ (Lmol^−1^cm^−1^)	ε_405_ (Lmol^−1^cm^−1^)	λ_em_ (nm)	E_gap_ (eV)	ϕ
TPO-L	371	215	193	78	448	4.74	0.12
TPOL-1	372	288	266	98	425	4.64	0.07
TPOL-2	372	287	265	103	426	4.70	0.07
TPOL-3	372	245	224	83	427	4.70	0.15

ε: molar absorptivity; λ_em_: emission maximum; E_gap_: HOMO–LUMO energy difference; ϕ is the quantum yield of photolysis. All data were obtained in acetonitrile.

**Table 2:** Molecular frontier orbital map of TPOLs and TPO-L.

PIs	Optimized Structures	HOMO	LUMO
TPO-L	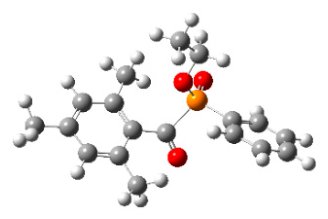	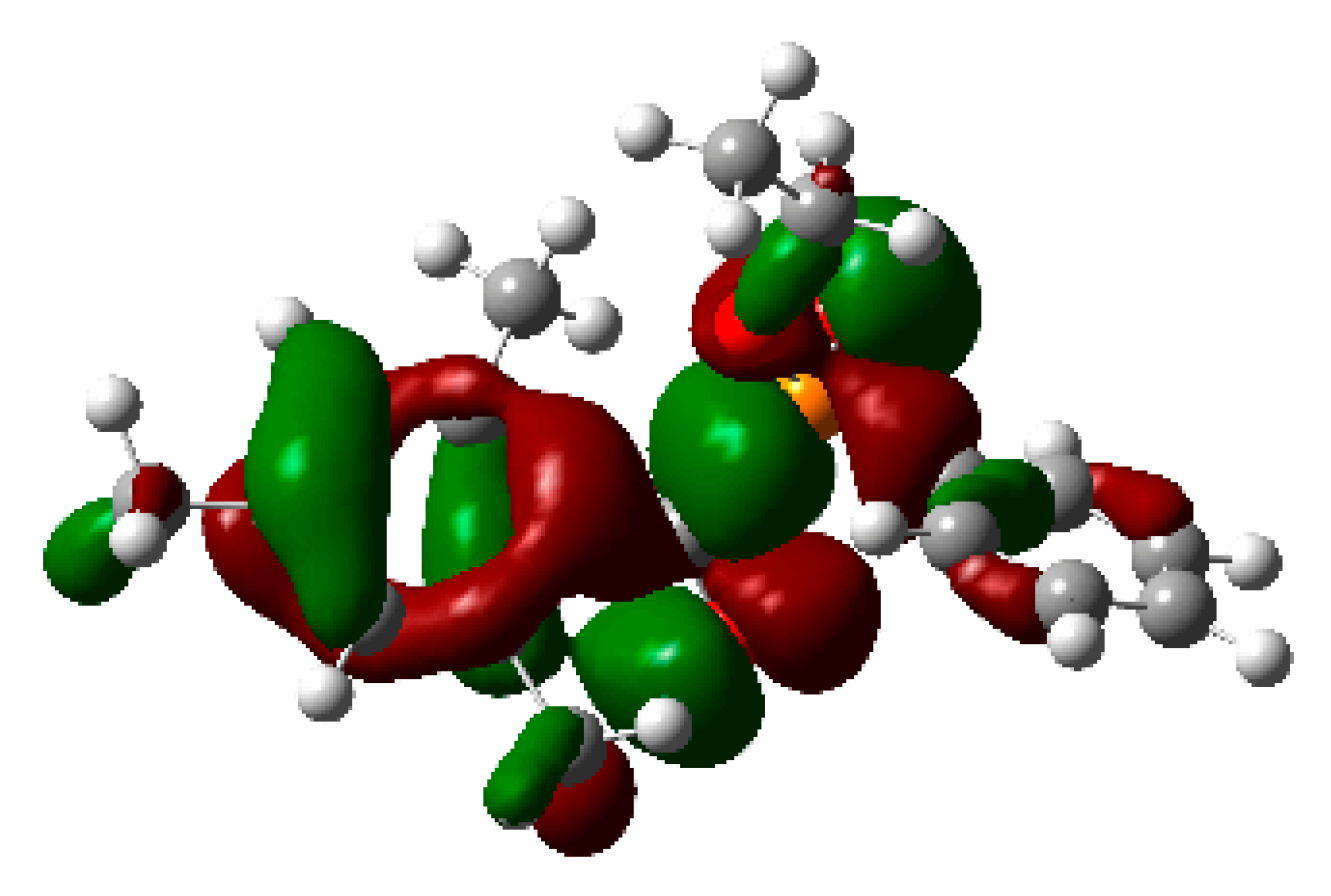 E = −6.43 eV	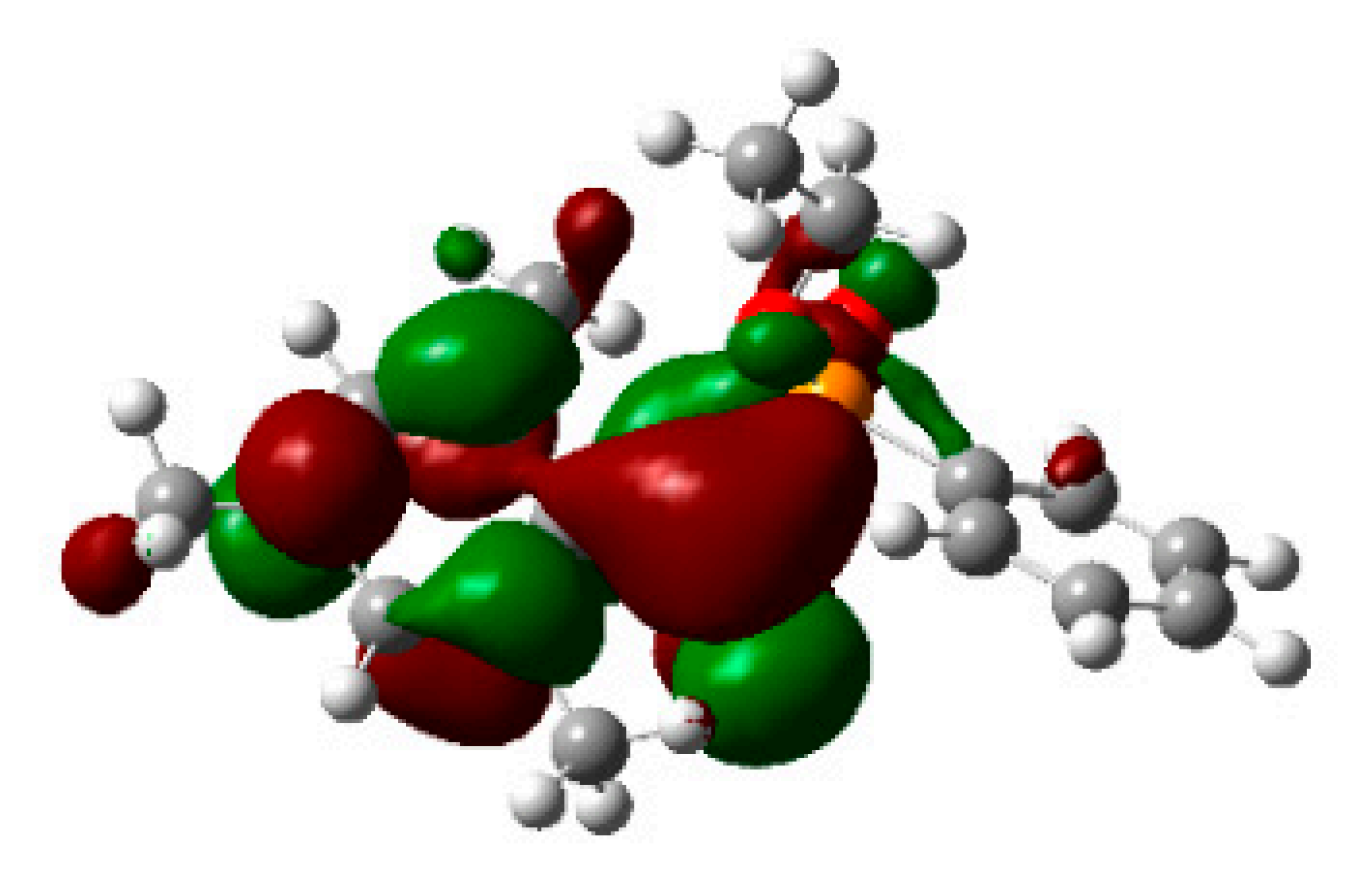 E = −1.69 eV
TPOL-1	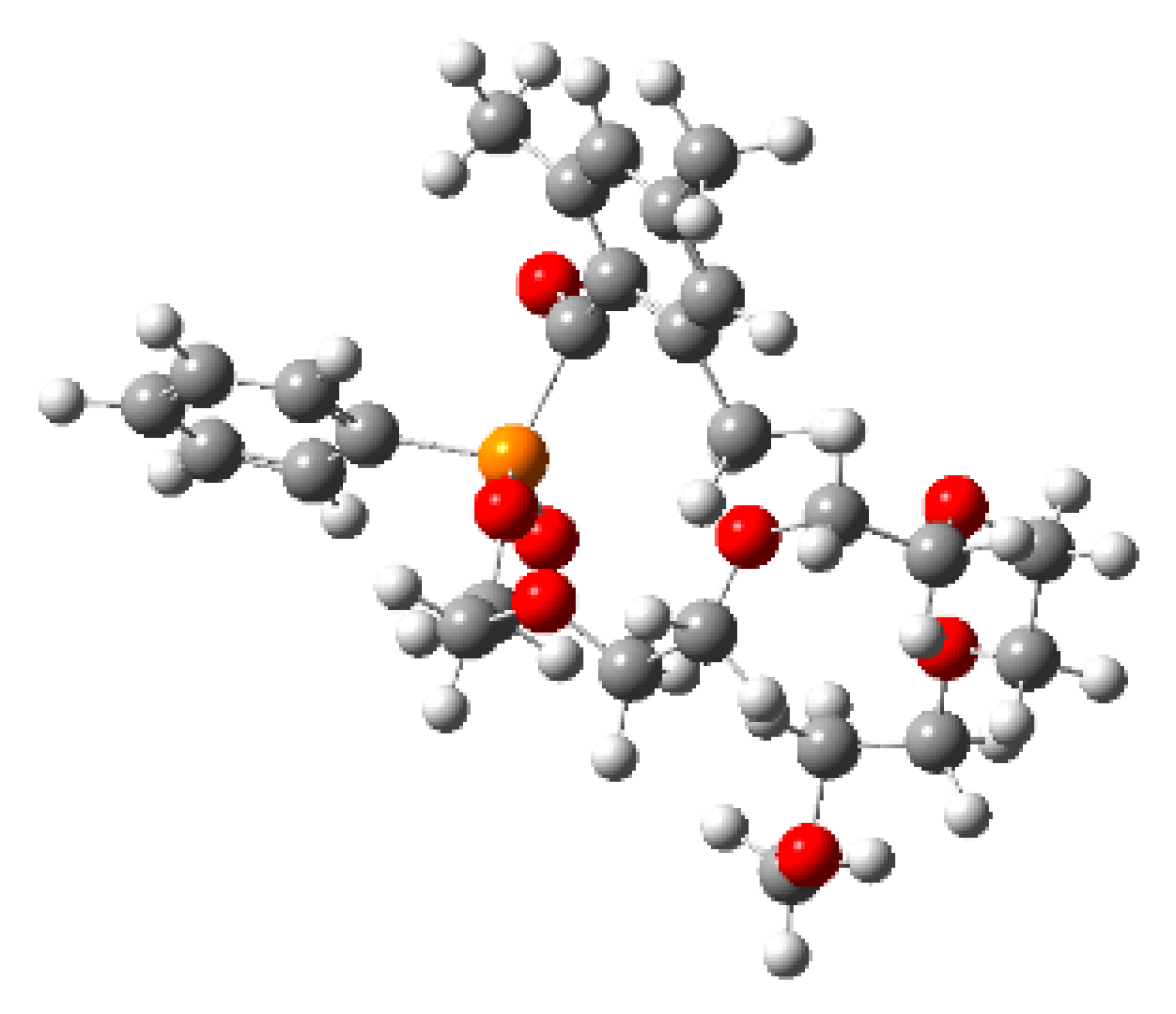	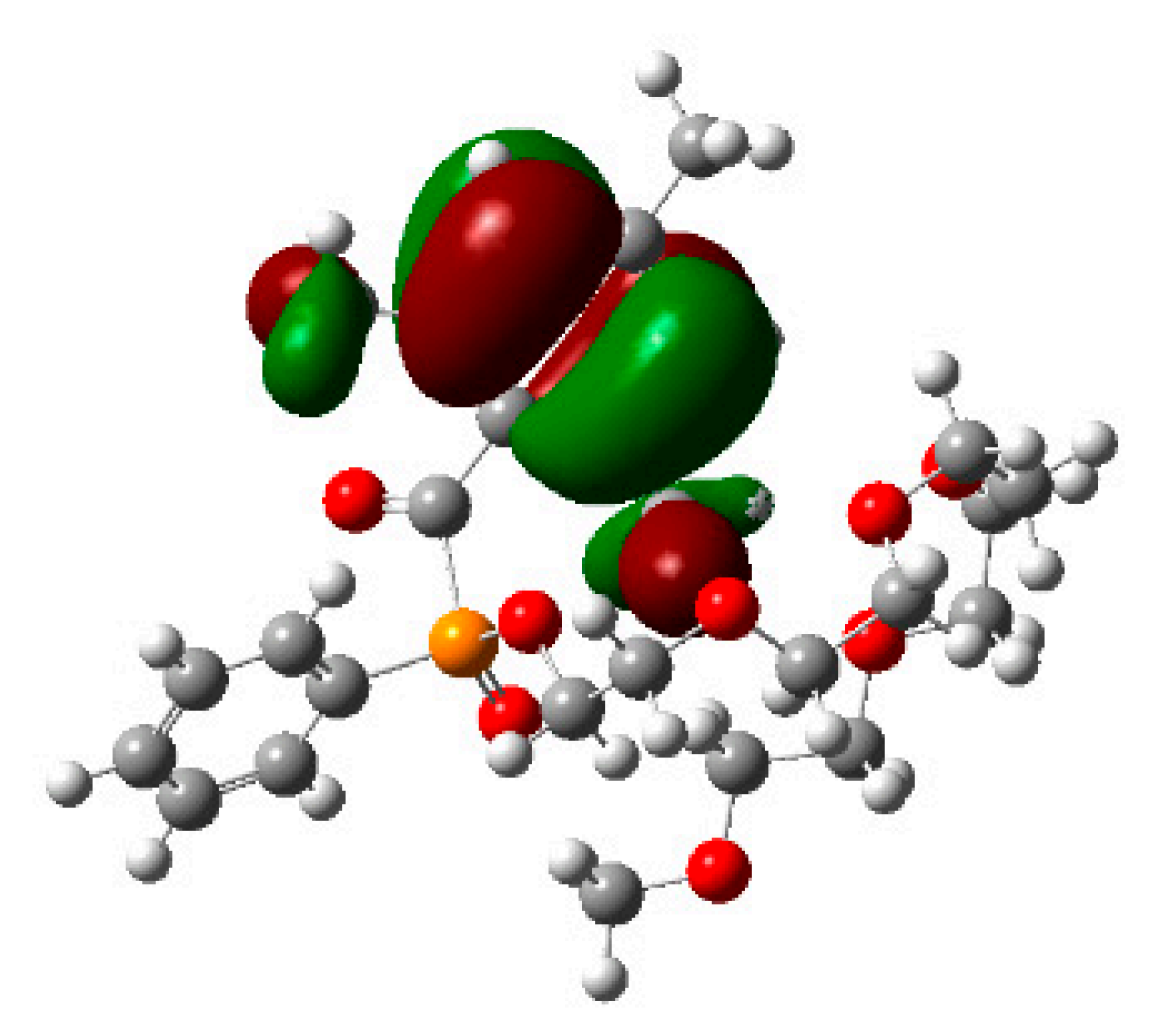 E = −6.1753 eV	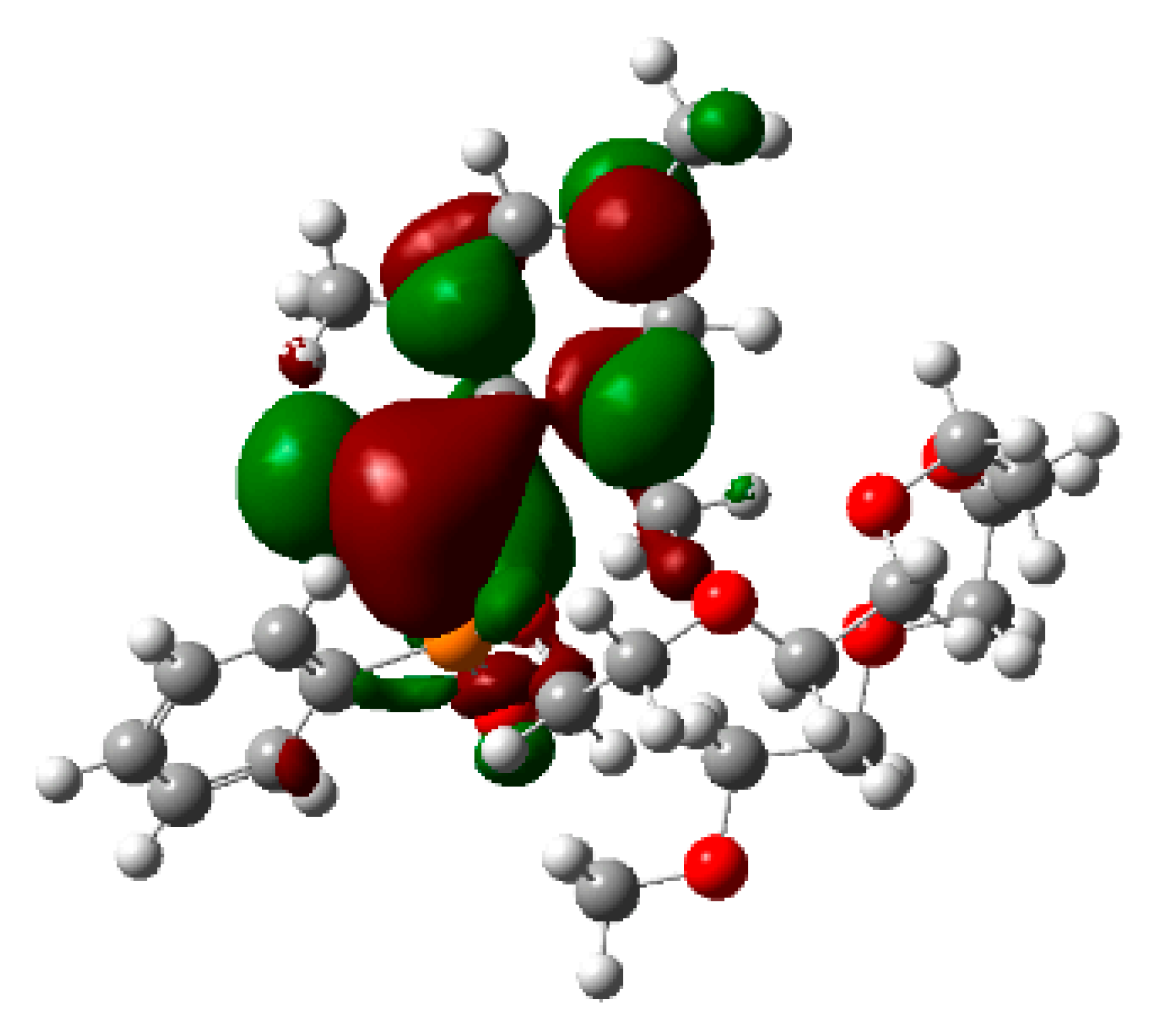 E = −1.5376 eV
TPOL-2	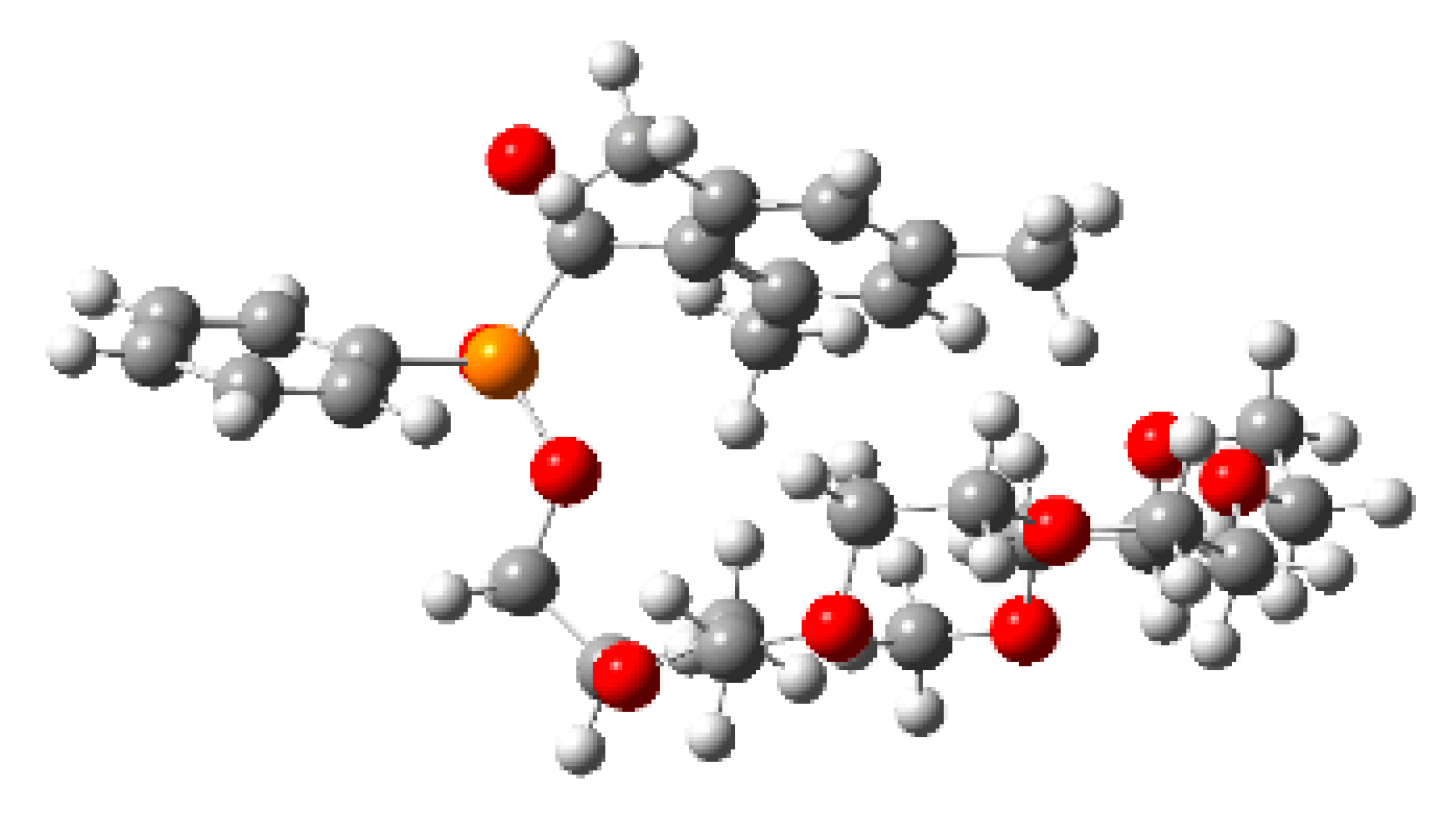	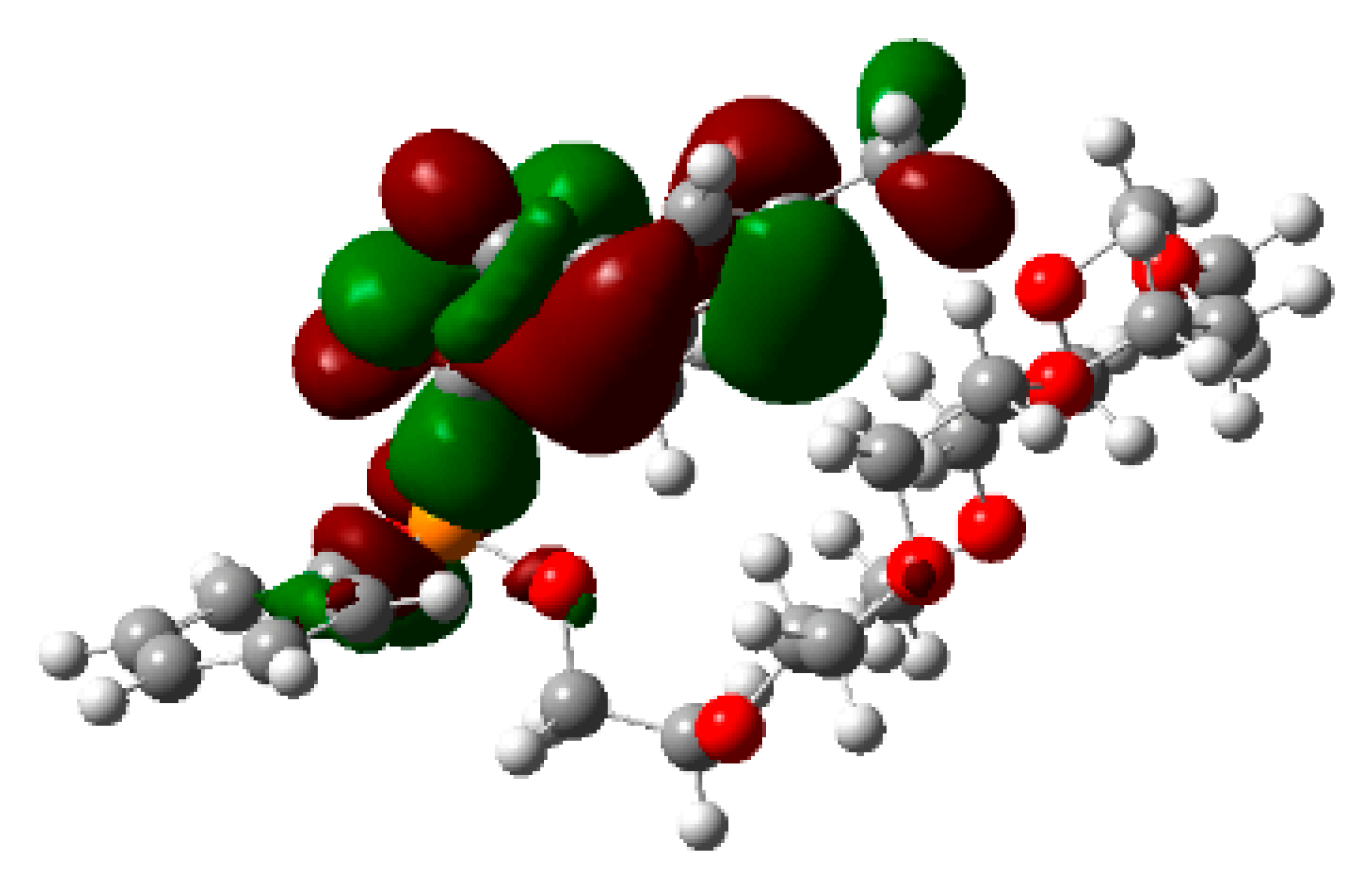 E = −6.3508 eV	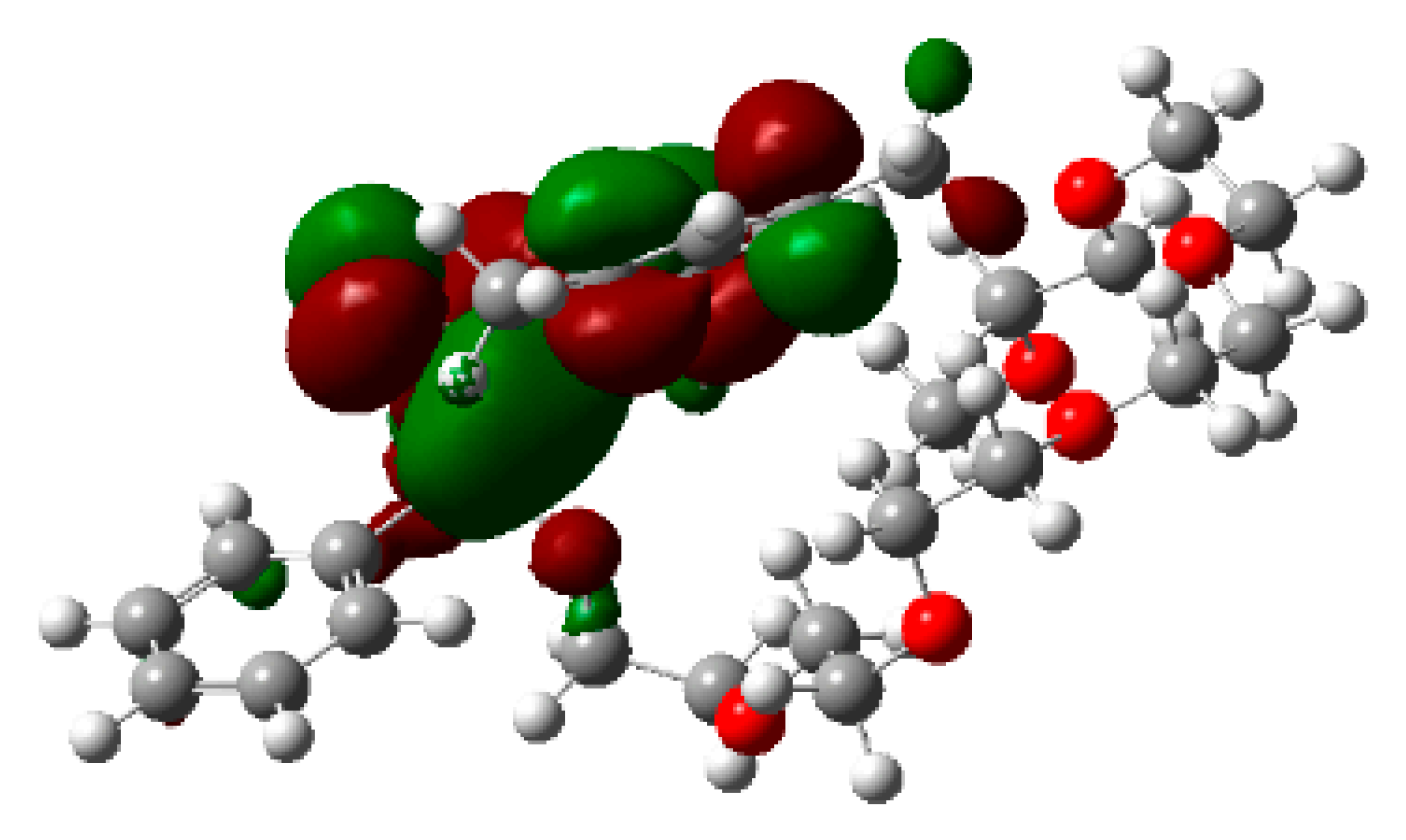 E = −1.6487 eV
TPOL-3	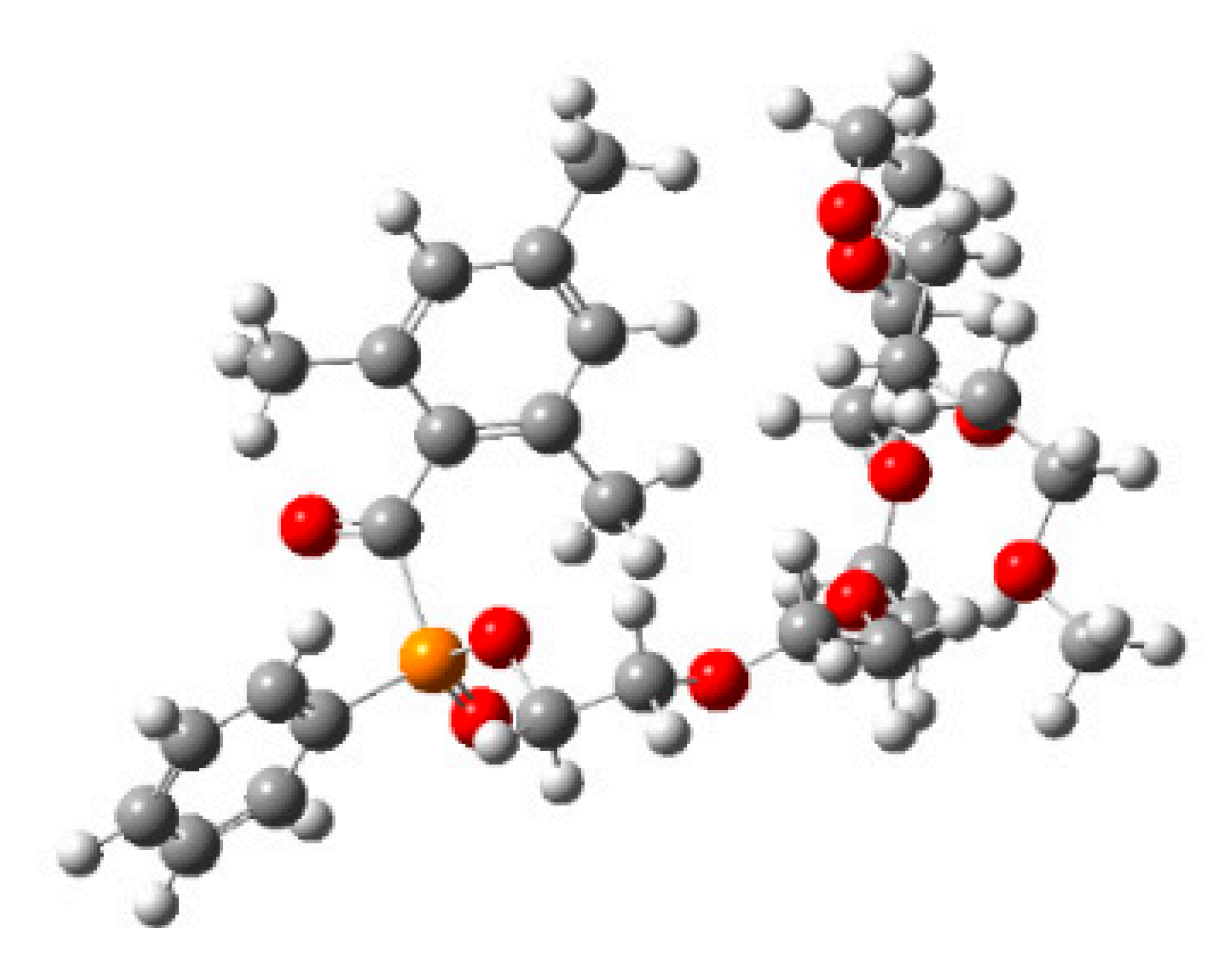	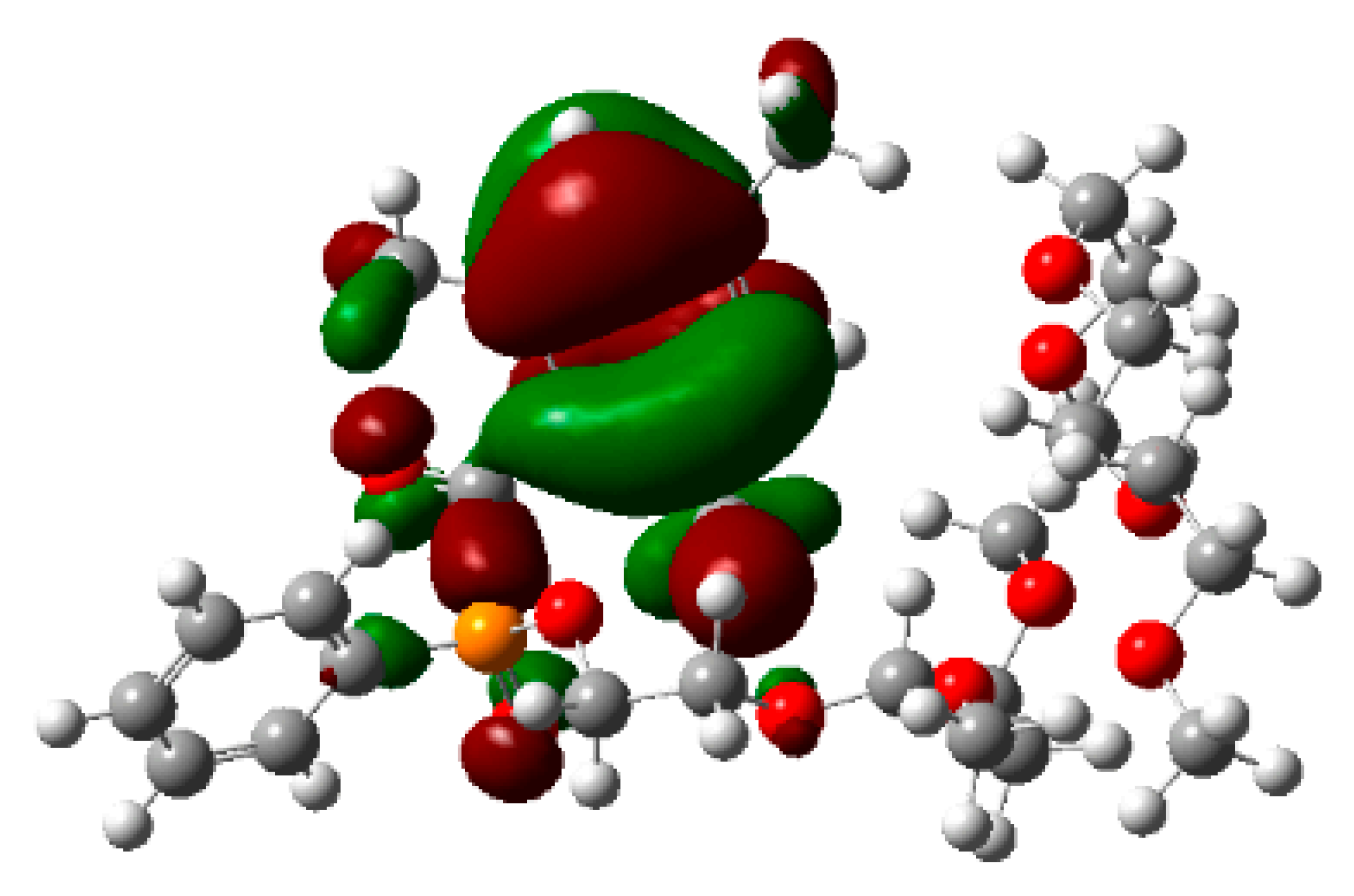 E = −6.0439 eV	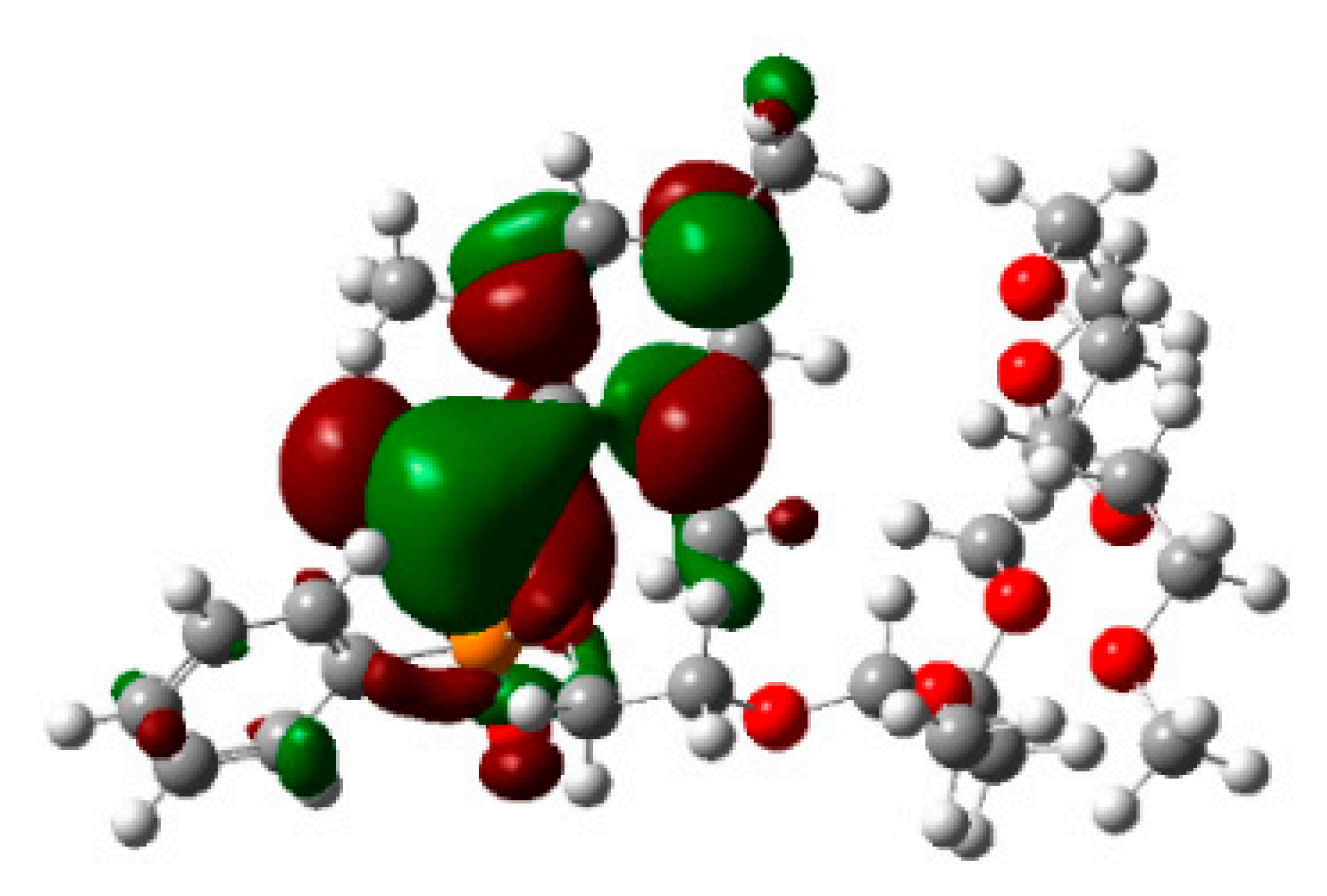 E = −1.3436 eV

**Table 3:** Migration rates of TPOLs.

PIs	M_r_ (g/mol)	λ_max_ (nm)	ε_max_ (Lmol^−1^cm^−1^)	A (a.u)	m (mg)	Migration Ratio
TPOL-1	521.38	370	288	0.0059	764.8	0.70%
TPOL-2	565.264	370	287	0.0010	711.1	0.14%
TPOL-3	610.29	370	245	0.0001	630.5	0.02%
TPO-L	316.34	370	215	0.0085	630.5	0.98%

The migration rate data were obtained in acetonitrile solution.

**Table 4:** Solubility of TPOLs.

PIs	S_TMPTA_ (wt%)	S_HDDA_ (wt%)	S_HPMA_ (wt%)
TPOL-1	>50	>50	>50
TPOL-2	>50	>50	>50
TPOL-3	>50	>50	>50
TPO-L	>50	>50	>50

## Data Availability

The original contributions presented in this study are included in the article/Supplementary Materials. Further inquiries can be directed to the corresponding authors.

## Supplementary Materials

The following supporting information can be downloaded at https://www.mdpi.com/article/10.3390/polym18212720/s1, Figure S1: Proposed cleavage mechanism for TPO-L derivatives (TPOLs); Figure S2: ^1^H NMR spectrum of TPOL-1; Figure S3: ^13^C NMR spectrum of TPOL-1; Figure S4: ^31^P NMR spectrum of TPOL-1; Figure S5: Mass spectrum of TPOL-1; Figure S6: ^13^C NMR spectrum of TPOL-2; Figure S7: ^1^H NMR spectrum of TPOL-2; Figure S8: ^31^P NMR spectrum of TPOL-2; Figure S9: Mass spectrum of TPOL-2; Figure S10: ^1^H NMR spectrum of TPOL-3; Figure S11: ^13^C NMR spectrum of TPOL-3; Figure S12: ^31^P NMR spectrum of TPOL-3; Figure S13: Mass spectrum of TPOL-3.
