# Supplementary material for: Novel Acyl Phosphine Oxides as Low-Migration Type I Photoinitiators for Radical Polymerization
Source: Polymers (Basel). 2026 Nov 2;18(21):2720. doi: 10.3390/polym18212720 (PMC13766143; doi:10.3390/polym18212720)
Supplement: Supplementary file 1 [file polymers-18-02720-s001.zip › polymers-4527163-supplementary.pdf]

Novel acyl phosphine oxides as low-migration Type I photoinitiators for radical polymerization

supporting information

S1. Experimental Section

S1.1 Materials

The preparation of phenyl(2,4,6-trimethylbenzoyl) phosphinic acid was carried out in our lab. All raw materials, solvents, and monomers used in the experiments were purchased from Energy Chemical (China).

S1.2 Instruments

The chemical structures of the TPOLs were confirmed by means of NMR, HRMS, and FT-IR spectroscopy. The NMR spectra (if applicable) were acquired on a Bruker Avance III instrument operating at 500 MHz and 600 MHz with $CDCl_3$ as the solvent and TMS as the internal reference. High-resolution mass spectrometric analysis was carried out on a Thermo Fisher Q Exactive mass spectrometer, while infrared spectra were recorded on a Nicolet iS50 spectrophotometer.

S1.3 Photolysis and fluorescence experiments

Photolysis of the four photoinitiators (TPO-L, TPOL-1, TPOL-2, and TPOL-3) was performed in CH_3CN at a concentration of 2 mM, using a UV-2450 spectrophotometer to monitor the process under irradiation at 365 nm (intensity: 100 mW/cm^2) for various time intervals. Their fluorescence emission spectra were recorded on a Hitachi-2700 fluorescence spectrometer under the same concentration and solvent conditions， and the excitation wavelength is 380 nm.

S1.4 Theoretical calculations

The HOMO (the lowest unoccupied molecular orbital) and LUMO(the highest occupied molecular orbital) energies were computed using density functional theory (DFT) at the B3LYP/6-31G(d) level.

S1.5 Electron spinning resonance spin trapping measurement (ESR-ST)

ESR spin-trapping (ESR-ST) spectra were recorded on a Bruker EMX-plus spectrometer using sample solutions composed of 1 mM photoinitiator and 0.01 M PBN dissolved in tert-butylbenzene. Before spectral acquisition, each sample was degassed with nitrogen for 5 min and subsequently exposed to 365 nm LED irradiation (100 mW/cm^2) for 1 min at ambient temperature. The proposed α-cleavage pathway is depicted in Figure S1.

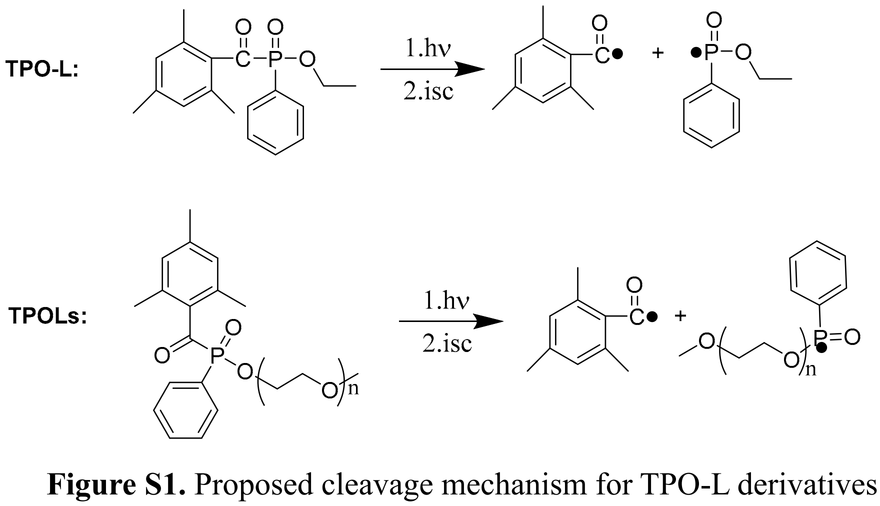

Figure S1. Proposed cleavage mechanism for TPO-L derivatives (TPOLs)

S1.6 Photopolymerization

Photopolymerization behaviors of photopolymerizable formulations composed of photoinitiators (TPO-L, TPOL-1, TPOL-2, TPOL-3) and trimethylolpropane triacrylate (TMPTA) monomer were investigated via a Nicolet iS50 Fourier-transform infrared (FT-IR) spectrometer. The concentration of the photoinitiator in the

photopolymerization system is 3.16 mmol per 100g TMPTA. In the first step, the formulation was coated onto a transparent polypropylene (PP) film using a 25 μm wire bar to give a wet film thickness of 25 μm. A second transparent PP film was then placed over the coating. After the upper PP film had fully conformed to the coating, the sandwiched sample was placed in the RT-FTIR optical path. Subsequently, the samples were exposed to LED@365 nm and LED@405 nm at a temperature of 25°C, with the light intensities being 100 mW·cm^{-2} and 50 mW·cm^{-2} respectively. Real-time FT-IR monitoring was initiated, and after a 5 second baseline acquisition, UV-LED irradiation was triggered. The variation in the C=C double bond absorption peak was continuously recorded over a 120 second irradiation period to track the photopolymerization process. The double bond conversion (DC) at the final stage of photopolymerization after irradiation time t was determined by Equation (S1):

$$\mathrm{DC} = \left(1 - \frac{A_{t(C=C)}/A_{t(C=O)}}{A_{0(C=C)}/A_{0(C=O)}}\right) \times 100\% \tag{S1}$$

In this equation, A_0(C=C) and A_0(C=O) are the peak areas of the corresponding functional groups measured prior to irradiation, and A_t(C=C) and A_t(C=O) are the peak areas determined at irradiation time t.

S1.7 Solubility

Equation (S2) was used to calculate the solubility of TPOLs and TPO-L in functional acrylate monomers at 25 °C:

$$S = \frac{m_{PI}}{m_{PI} + m_{AM}} \times 100\% \tag{S2}$$

In this equation, S stands for the solubility of the photoinitiator (PI), m_{PI} represents the mass of the photoinitiator, and m_{AM} is the mass of the acrylate monomer.

S1.8 Migration

Photopolymerization of TMPTA formulations containing 2 wt% of TPO-L or TPOLs was carried out in a nitrogen-purged erlenmeyer flask upon 365 nm LED irradiation (100 mW·cm^{-2}) for 6 min. The cross-linked polymers thus obtained were ground into small pieces and soaked in acetonitrile (CH_3CN) at 25 °C for 24 h. The resulting solution was filtered, and its UV-vis absorption spectra were recorded. The amount of PI leached from the polymer network was then calculated from the absorbance of the

photoinitiator (PI) at its maximum absorption wavelength using Equation (S3):

$$\text{migration ratio} = \frac{A \times M_r \times V}{\varepsilon \times l \times m_0} \times 100\% \qquad \text{(S3)}$$

In this equation, A is the absorbance of the photoinitiator (PI) at its maximum absorption wavelength, M_r is the molecular weight of PI, V is the volume of the filtrate, ε is the molar extinction coefficient of PI at the absorption maximum, l is the optical path length (1 cm), and m_0 is the photoinitial mass of PI incorporated into the photopolymerizable system.

S1.9 Cytotoxicity test

The cytotoxicity of TPOL-1, TPOL-2, TPOL-3 and TPO-L, was evaluated using mouse fibroblast L929 cells. L929 cells were cultured in basal medium consisting of 89% MEM (Corning, 10-010-CV), 10% fetal bovine serum (Procell, 164210-50), and 1% penicillin–streptomycin (Biosharp, BL505A) at 37 °C under 5% CO_2. Briefly, L929 cells were seeded into 96-well plates at a density of 8×10^3 cells per well and cultured for 24 h. Briefly, L929 cells were seeded into 96-well plates at a density of 8×10^3 cells per well, and 100 μL of photoinitiator-containing medium at different concentrations (5, 25, and 50 μM) was added to each well, followed by incubation for 24 h. After removal of the culture medium, the wells were washed three times with PBS(Procell, PB180327). Then, medium containing 10% CCK-8 (Invigentech, IV08-100) was added to each well, and the plates were incubated at 37 °C under 5% CO_2 for 2 h. The absorbance was measured at 450 nm using a microplate reader to obtain the cytotoxicity data.

S1.10 Quantum yield of photolysis

The quantum yield (ϕ) of photoinitiators after α-cleavage was measured by steady-state irradiation in air-saturated acetonitrile solution. The photon quantum yield of TPO was measured in ref. 30 (ϕ TPO=0.56) [36]. The photon quantum yield of the new photoinitiator is calculated by the following equation (S4):

$$\Phi_{sample} = \Phi_{TPO}\, [\varepsilon_{TPO}\, slope_{sample}] / [\varepsilon_{sample}\, slope_{TPO}] \qquad \text{(S4)}$$

Where, Slope is the slope of the function ln [exp ($2.3OD_{370nm}$) −1] = f(t), OD is the absorbance of photoinitiator at different irradiation times with @LED365nm [37].

S2 NMR spectra and mass spectra of molecular structures

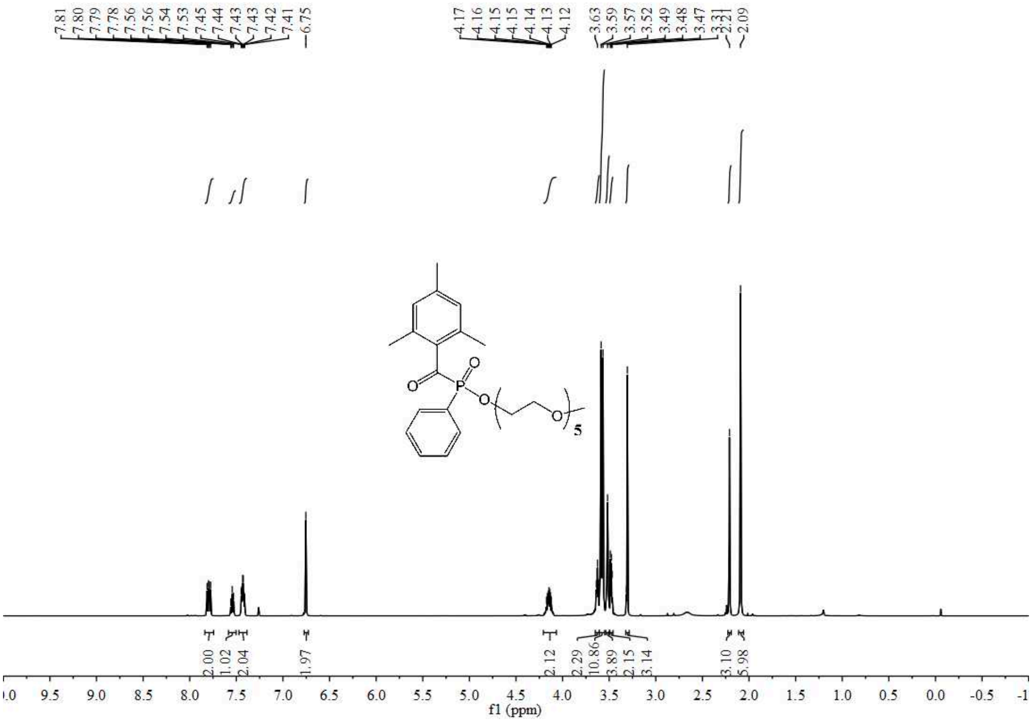

Figure S2. ^{1}H NMR spectrum of TPOL-1

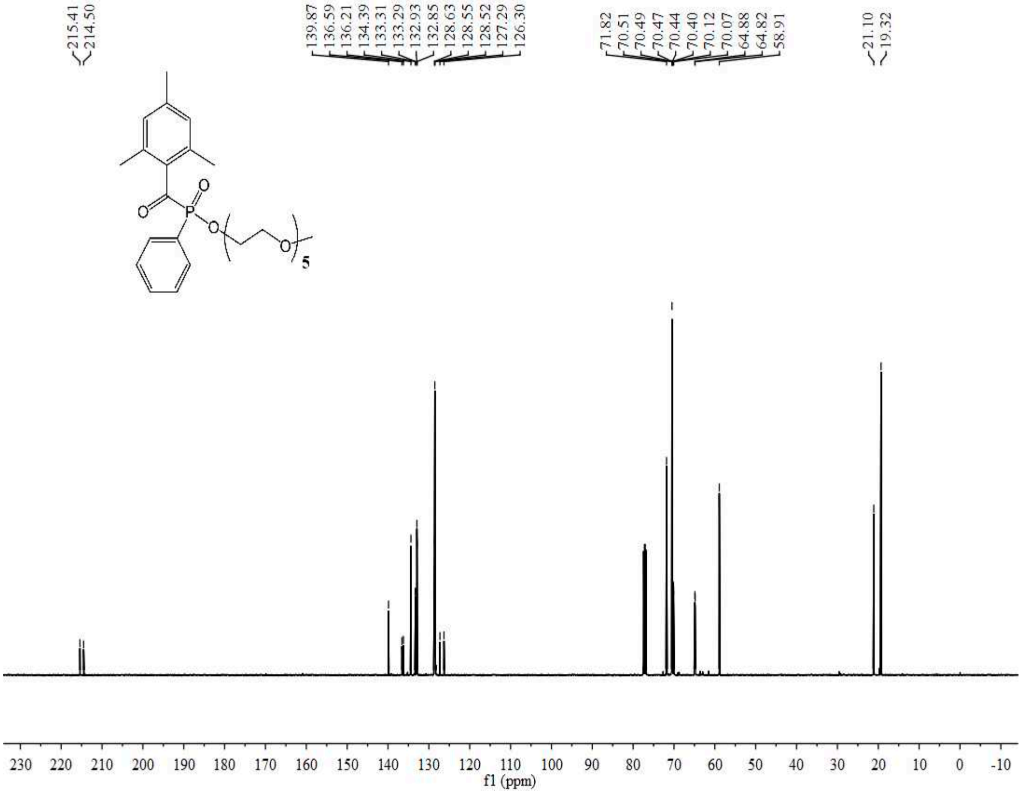

Figure S3. ^{13}C NMR spectrum of TPOL-1

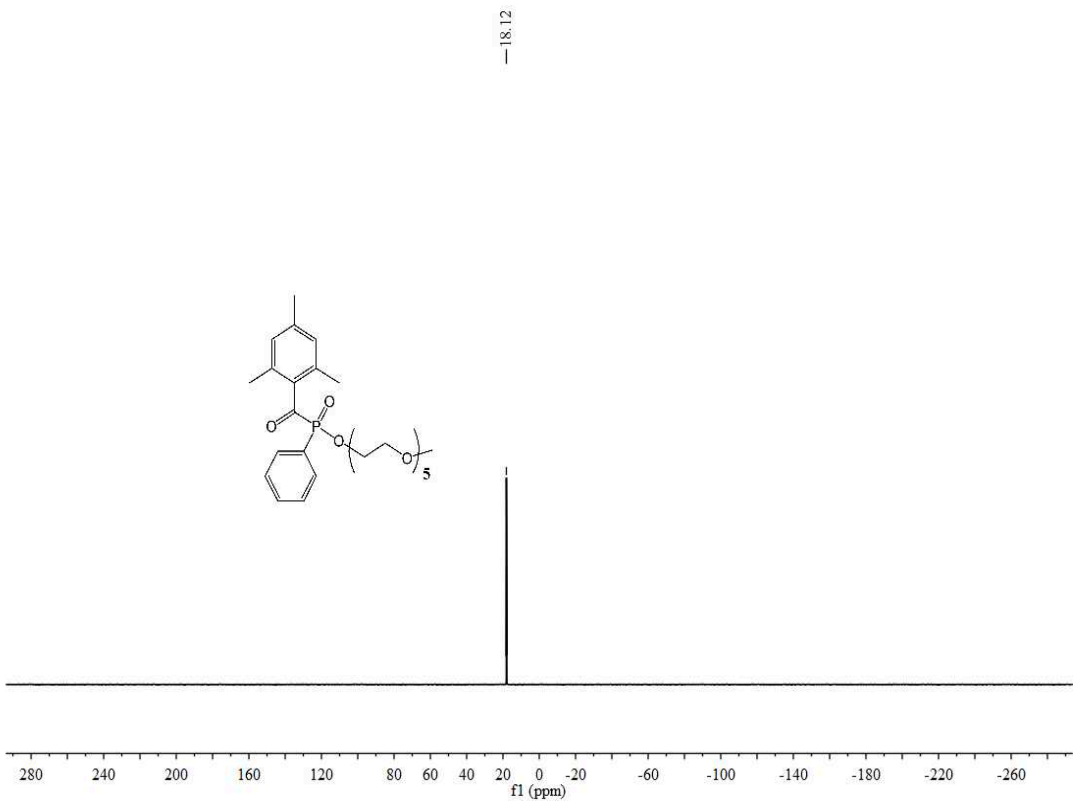

Figure S4. ^{31}P NMR spectrum of TPOL-1

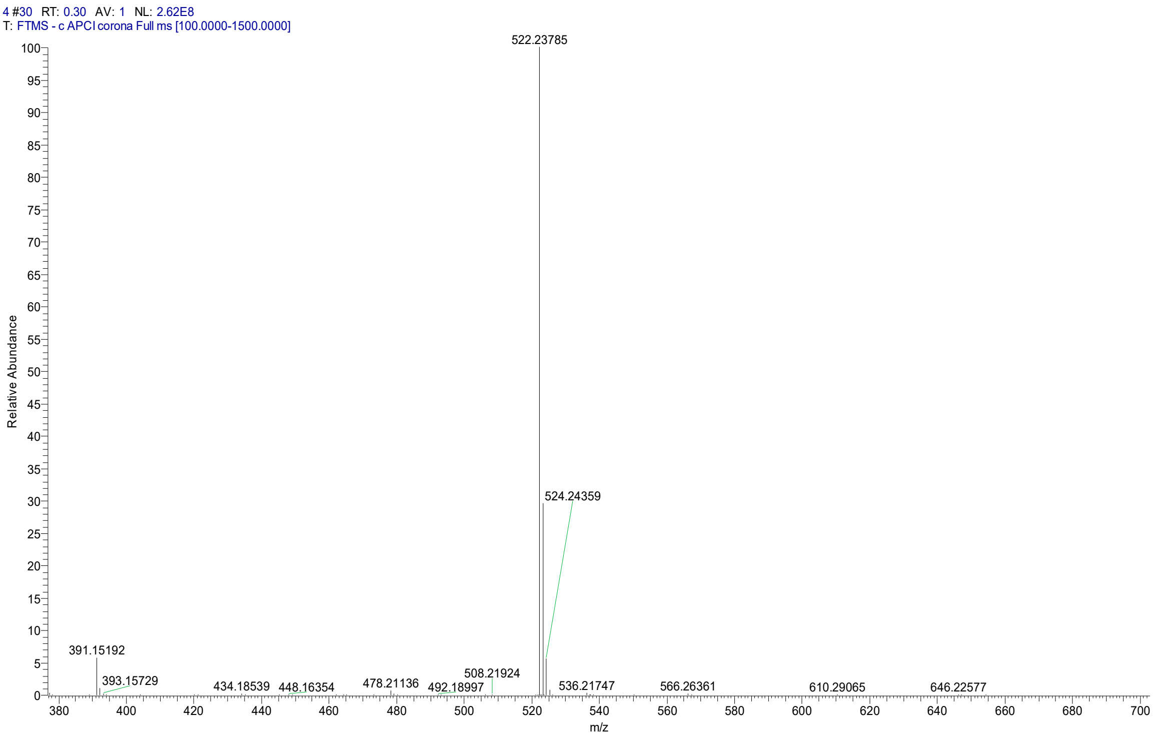

Figure S5. Mass spectrum of TPOL-1

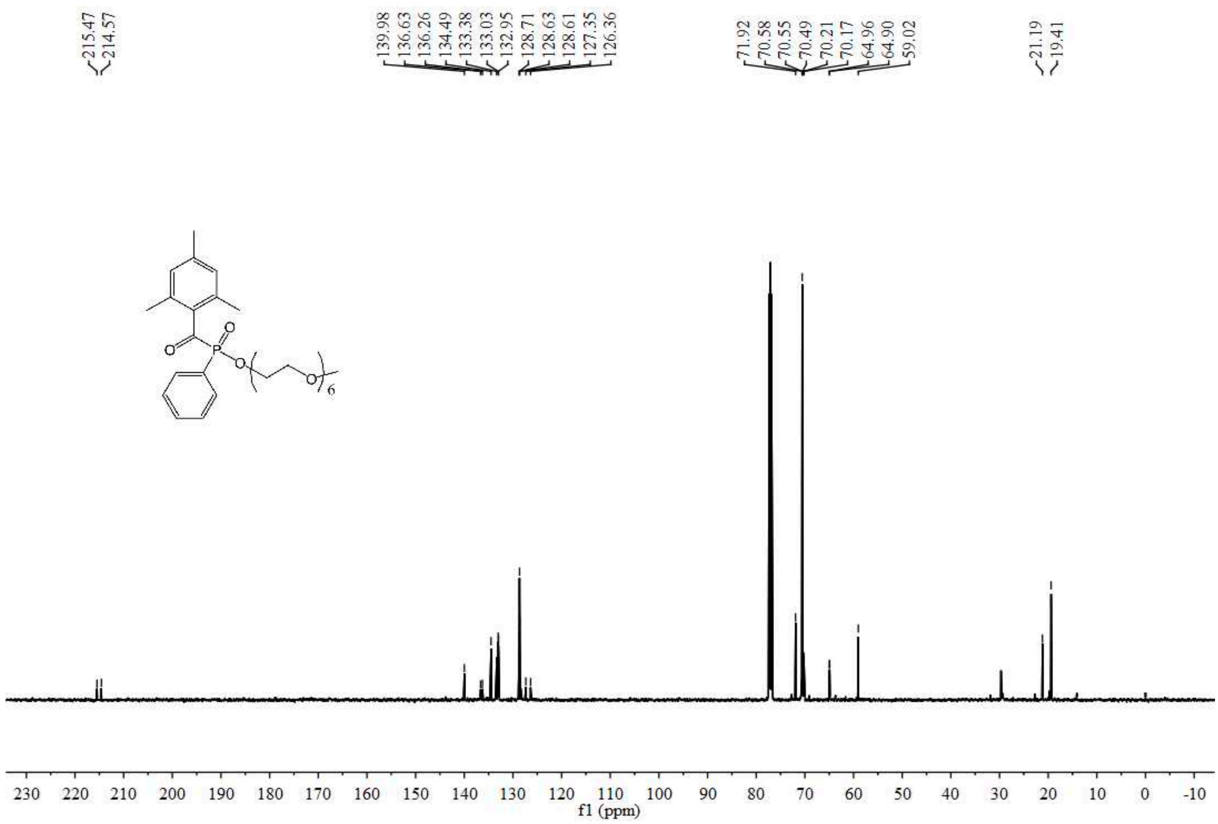

Figure S6. ^{13}C NMR spectrum of TPOL-2

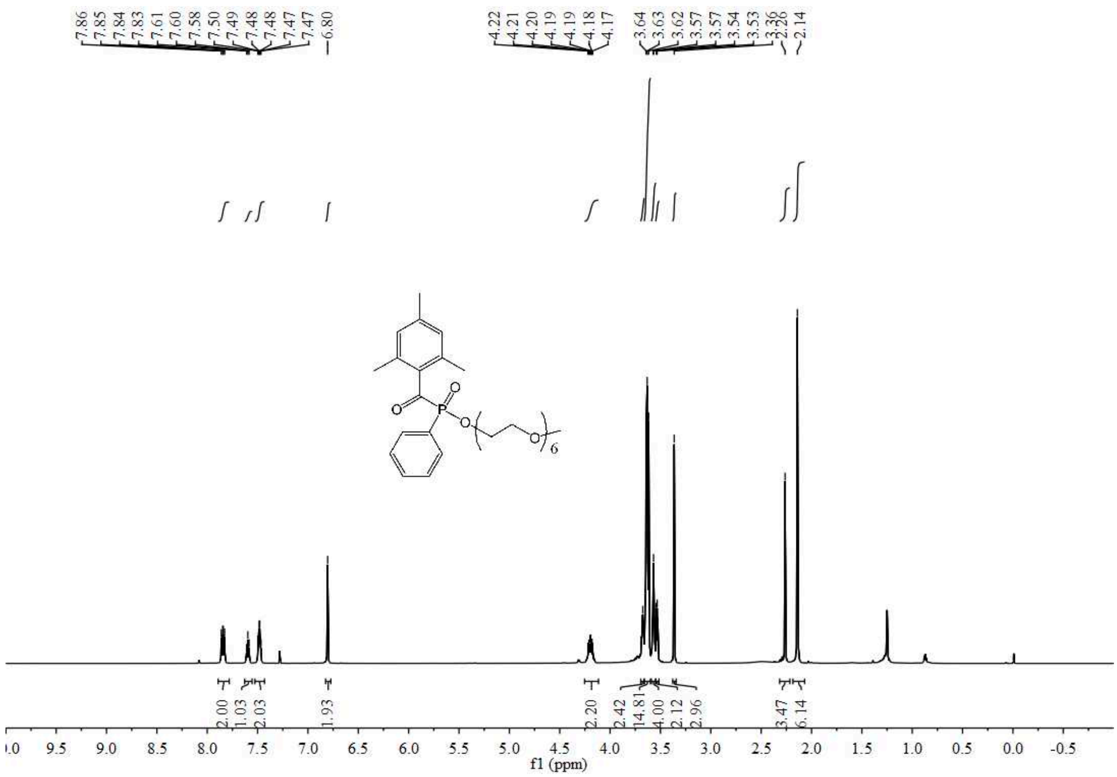

Figure S7. ^{1}H NMR spectrum of TPOL-2

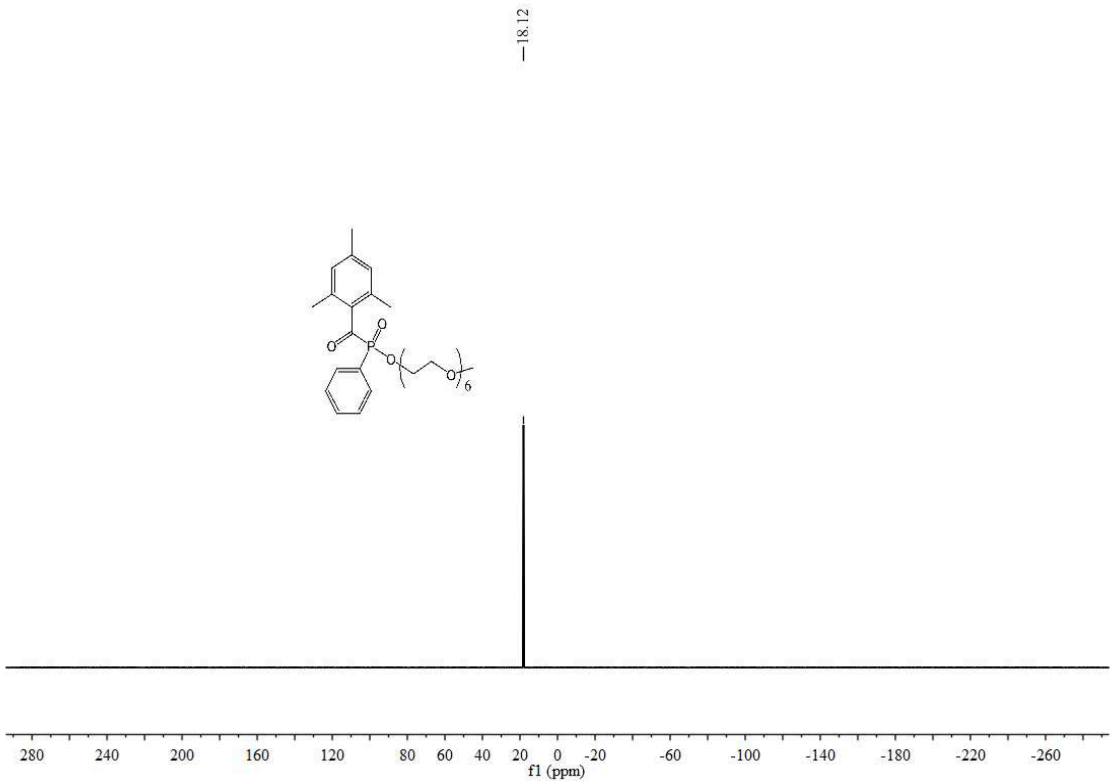

Figure S8. ^{31}P NMR spectrum of TPOL-2

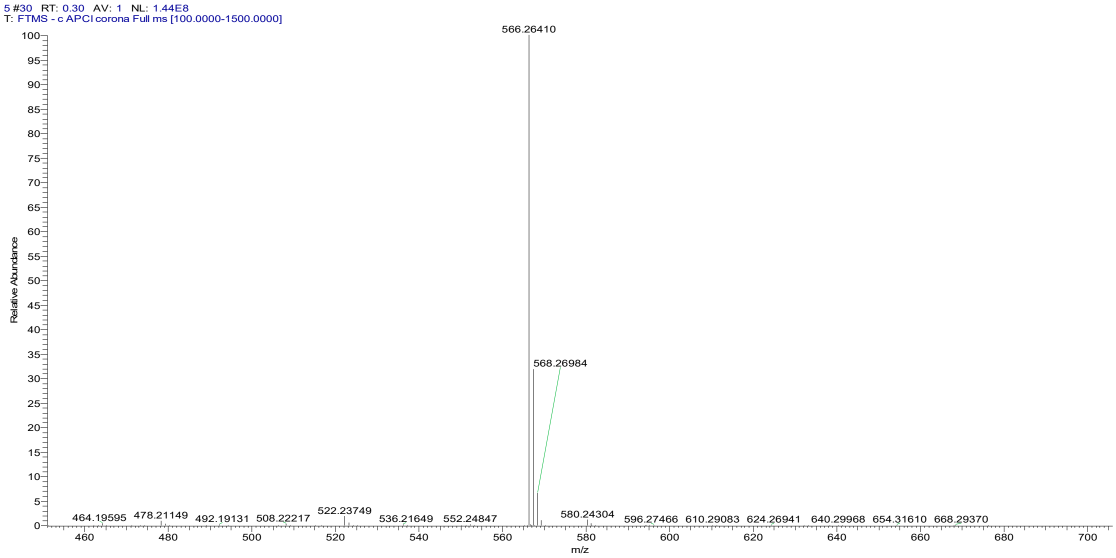

Figure S9. Mass spectrum of TPOL-2

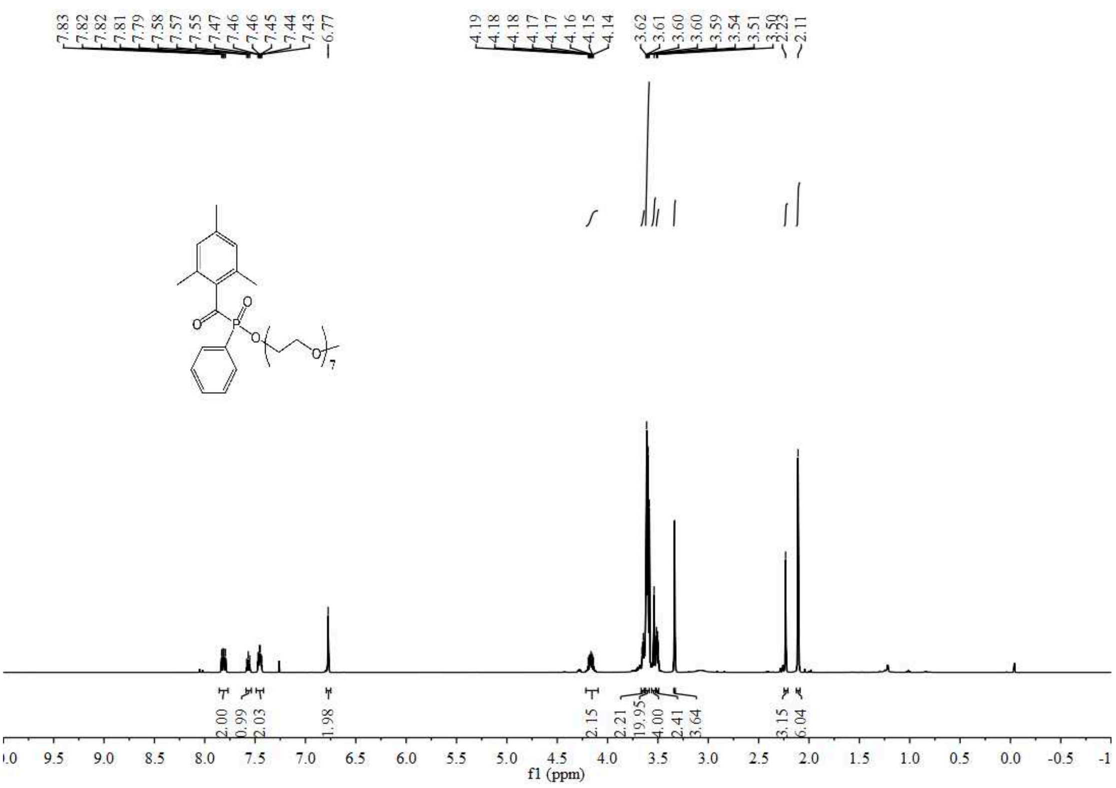

Figure S10. ^{1}H NMR spectrum of TPOL-3

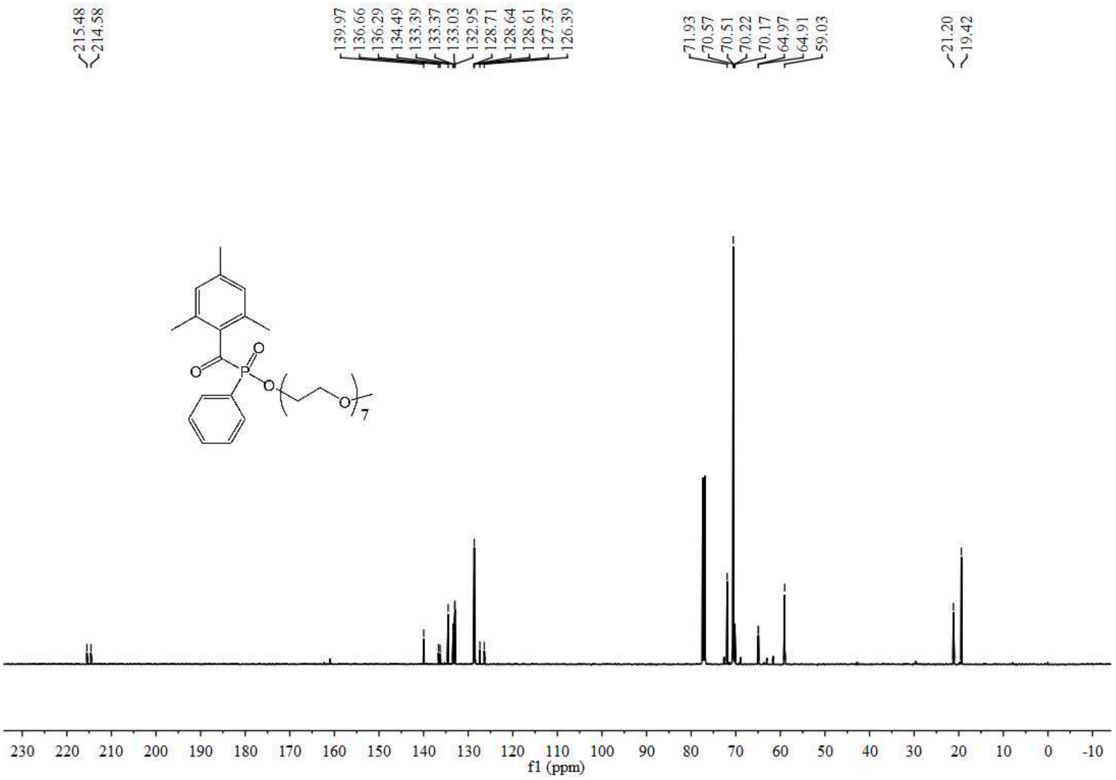

Figure S11. ^{13}C NMR spectrum of TPOL-3

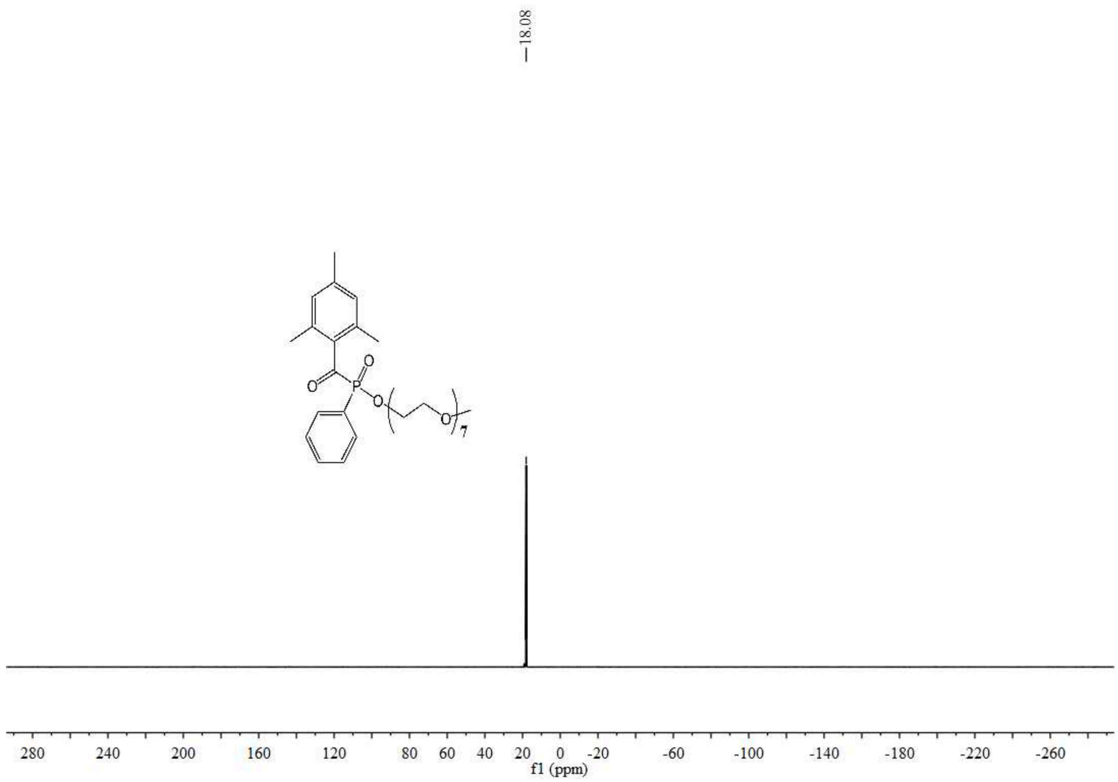

Figure S12. ^{31}P NMR spectrum of TPOL-3

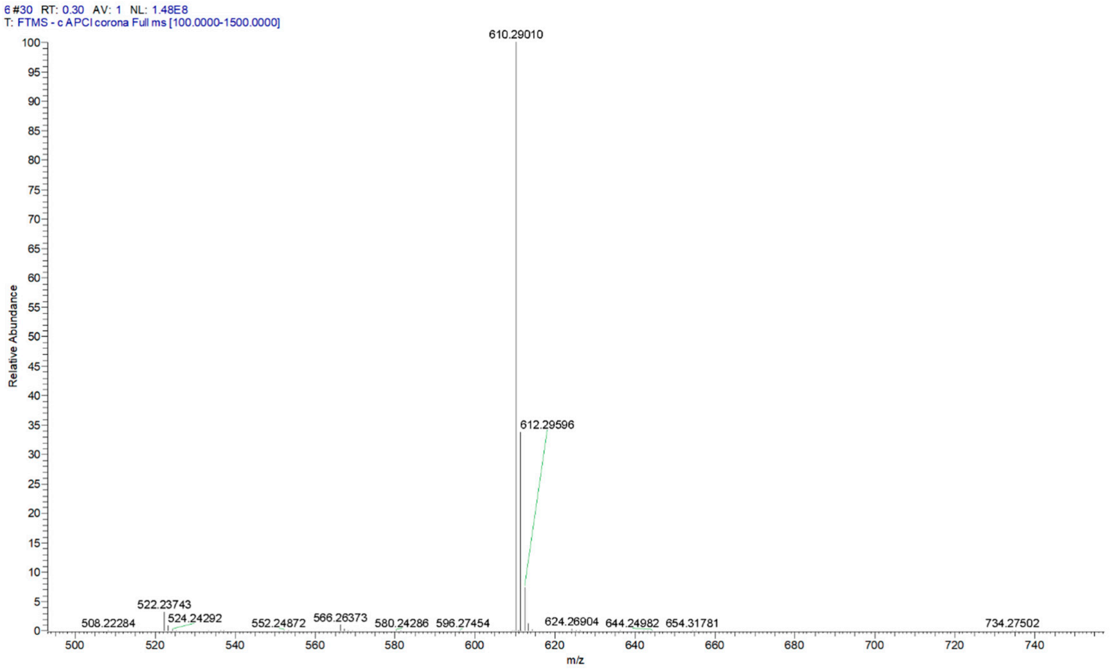

Figure S13. Mass spectrum of TPOL-3
